# Sensorimotor tests in patients with neck pain and its associated disorders: a systematic review and meta-analysis

**DOI:** 10.1038/s41598-024-63545-3

**Published:** 2024-06-04

**Authors:** Niklas Särkilahti, Milka Hirvonen, Joona Lavapuro, Jani Takatalo, Eliisa Löyttyniemi, Olli Tenovuo

**Affiliations:** 1 Department of Clinical Neurosciences, Faculty of Medicine, University of Turku, Turku, Finland; 2 Medical Research Center Oulu, University of Oulu, Oulu, Finland; 3 Loisto Terveys, Oulu, Finland; 4 The Department of Biostatistics, University of Turku and Turku University Hospital, Turku, Finland; 5 Turku Brain Injury Centre, Turku University Hospital, Turku, Finland; 6 Neurocenter, Turku University Hospital, Turku, Finland; 7 Faculty of Medicine, University of Turku, Turku, Finland

**Keywords:** Medical research, Bone quality and biomechanics, Pain

## Abstract

This systematic review aimed to synthesize the current evidence regarding neck sensorimotor testing in individuals with neck pain, assess the differences between neck pain groups and healthy controls, and recognize factors that might affect test results. We performed the data search using PubMed, Embase, PsycINFO, CINAHL, and Scopus databases. We used a two-step screening process to identify studies. Furthermore, we screened the reference lists for additional studies. Hedges g was used to present the difference between neck pain groups and asymptomatic individuals. We assessed the quality of the studies using the QUADAS tool. The final review included 34 studies, of which 25 were related to the joint position error test, four to the smooth pursuit neck torsion test and six to the balance test. Our meta-analysis showed poorer joint-position sense, oculomotor function, and wider postural sway in individuals with neck pain than healthy controls. The size of the difference between the groups seemed to be influenced by the intensity of the pain and the presence of dizziness. Therefore, it might be helpful in future studies to differentiate patients with neck pain into subgroups based on their symptom and demographic profiles to assess other factors that significantly affect cervical sensorimotor control.

## Introduction

Sensorimotor control is defined as the central nervous system’s control of movement, balance, posture, and joint stability^1,2^. To maintain functional joint stability and generate adaptable, efficient, and complex motor actions, the central nervous system collects information from all peripheral sensory systems, such as the visual, auditory, vestibular, and proprioceptive systems^1–3^. Deficits in sensorimotor control, such as the reduced ability to reproduce previous predetermined cervical positions (joint position sense) and increased postural instability in standing, have been shown in individuals with neck pain^4–8^. These deficits have been suggested to be related to altered cervical proprioception due to pain, effusion, trauma or fatigue^1^. Therefore, sensorimotor control tests such as joint position sense, movement control, balance and oculomotor tests and exercises are commonly used in clinical practice in patients with neck pain. However, opposite results have also been reported. De Zoete et al.^9,10^, for example, did not find a difference in seven cervical sensorimotor control tests between the neck pain group and those without symptoms, nor a clear relationship between sensorimotor control and pain or disability.

The reason for the inconsistent results has remained unclear. De Zoete et al.^9^ speculated that the conflicting results may be due to different recruitment methods of the study group or biases such as small sample size and lack of blinding. However, the literature has also indicated that different neck pain symptom profiles may affect sensorimotor control. For example, dizziness and the intensity and location of neck pain seem to influence the results of sensorimotor control tests^8,11–14^. The different symptom profiles may also explain why the results of individuals with neck pain within and between studies seem to vary significantly more than those without symptoms^6–8^.

The sensitivity and specificity of the used tests are crucial for correct patient classification and treatment targeting. Inconsistent and variable results have challenged the conclusion that neck pain alone is the underlying cause of sensorimotor control deficits. However, sensorimotor control tests and treatments may still be indicated for some neck pain groups. Therefore, our systematic review aimed to synthesize the current evidence regarding sensorimotor testing in individuals with neck pain, assess the differences between different neck pain groups and healthy controls, and recognize factors that might affect test results.

## Methods

This systematic review and meta-analysis were registered prospectively on the International Prospective Register of Systematic Reviews (PROSPERO) database (registration number: CRD42020207504). Reporting was done in line with Preferred Reporting Items for Systematic and Meta-Analyses (PRISMA)^15^.

### Search strategy

We performed the data search using PubMed, Embase, PsycINFO, CINAHL, and Scopus databases published from inception to the date of search, October 17th, 2020. We updated the search on May 9th, 2023. With support from the university librarian, we developed the strategy, including Medical Subject Headings and free-text terms and adapted it to the search language of each database. Our search strategy used three groups of keywords: neck pain, sensorimotor tests, and diagnostic test indicators. The complete search strategy is shown in detail in Appendix 1. We also manually screened the reference lists of the included studies for additional studies.

### Article selection

We used a two-step screening process to identify studies. Initially, the two evaluators (NS and MH, in the updated search NS and JL) independently reviewed the titles and abstracts of the studies and graded the studies as ‘potentially relevant’ or ‘insignificant’. In the second phase, the evaluators independently performed a full-text review of the studies identified as ‘potentially relevant’ and graded them as ‘relevant’ or ‘insignificant’. In both phases, the evaluators met to discuss their study selections and to resolve disagreements. Another evaluator (JT) made the decision if no consensus was found.

An article was included if it met the following criteria: (1) a full-text original article; (2) published in English in a scientific peer-reviewed journal; (3) adult (≥ 18 years old) patients with neck pain and/or healthy individuals; (4) the reliability and/or validity of the sensorimotor test is assessed. All study designs were included in the review. The exclusion criteria were: (1) recommendations, comments, dissertations, reports, conference proceedings, treatment recommendations, books or book articles, and lecture materials; (2) literature reviews; (3) a concurrent condition that could affect the nervous system (e.g., multiple sclerosis, Parkinson's disease) or vestibular system (e.g., Meniere's disease, benign paroxysmal positional vertigo) present.

### Quality assessment

Two reviewers (NS and JL) independently applied the Quality Assessment of Diagnostic Accuracy Studies (QUADAS-2) to each study to evaluate the methodological quality^16^. We choose QUADAS-2 because it is recommended for systematic reviews to assess the risk of bias and applicability of diagnostic accuracy studies^17^. The QUADAS-2 comprises four domains: patient selection, index test, reference standard, and flow and timing, rated on a 3-point scale concerning the risk of low, high, or unclear bias^17^. However, we modified the tool to be more suitable for our research. We assumed that most studies included in the review have a case–control design. Therefore, we excluded this signalling question from the patient selection domain. In addition, we did not require the study to use both the index test and the reference standard. We evaluated whether standardized meter and implementation were used and, if not, whether the comparability of the conduct or interpretation of the index test to the reference standard was considered. We also assessed whether the testers were blinded to the subjects. We resolved mismatches by discussion. We included all selected articles in the study regardless of the risk of bias.

### Data extraction

Two reviewers extracted data from the original (NS and MH) studies and the updated (NS and JL) search. We extracted the same information from each study: sample size, sex, age, height, weight, duration of symptoms, symptom and functional capacity assessments (Visual Analogue Scale, Neck Disability Index, Tampa Scale of Kinesiophobia, etc.), description of the test and used instrument, and the results. After the data partition, it was organized according to the symptom profiles and tests by the first reviewer and checked by the second (MH) and third (JL) reviewers.

### Data synthesis and analysis

The inter-rater reliability between the evaluators’ gradings (‘relevant’ or ‘insignificant’) was calculated using percentages of agreement and Cohen Kappa with a 95% confidence interval (CI) at both screening stages. Kappa values above 0.81 have been proposed as almost perfect; 0.61–0.8 as substantial; 0.41–0.6 as moderate; 0.21–0.4 as fair; and below 0.2 as poor^18^. In addition, inter-rater disagreement was evaluated with McNemar’s test.

All the data from the studies were transferred to an Excel spreadsheet and divided according to each type of neck pain (e.g., non-specific neck pain, radiating neck pain, traumatic neck pain, cervicogenic dizziness). If the study only presented the results of one group or included different neck pain groups but did not segregate the results, the study was excluded.

Variables and descriptive data for each test were extracted and summarized using means and standard deviations (SD). Where standard error (SE) was only reported, the SD was calculated ($SD=SE \times \surd n$). Results presented merely as correlations, differences between groups or medians, or in figures were excluded. For descriptive data, the balance between gender distribution and studies was tested with Fisher’s exact test. P-values (two-tailed) less than 0.05 were considered statistically significant.

Data were analyzed when at least three studies reported the results of the same variable and parameter. Although in meta-analyses, the number of studies (at least five studies) instead of sample sizes is emphasized, as in the random effects modelling of meta-analyses^19–21^, the rule of three studies was chosen because we estimated that the data would otherwise remain small. Hedges g (random effect model) with a 95% CI was used to present the difference between different neck pain groups and between different neck pain groups and asymptomatic individuals, depending on the available data. The difference between groups was interpreted as small if the effect size (ES) was 0.2, as medium if the ES was 0.5, and as large if the ES was 0.8 or more^22^. In addition, a Z-test (with p-value) was used to assess the overall effect and I^2^ to evaluate the amount of statistical heterogeneity, with interpretation thresholds of low if I^2^ = 25%, medium if I^2^ = 50%, and high if I^2^ = 75%^23^.

EL performed all statistical analyses with SAS software, Version 9.4 of the SAS System for Windows (SAS Institute Inc., Cary, NC, USA).

### Dual publication

A short summary of this publication has previously been published at the University of Turku webpages at https://www.utupub.fi/handle/10024/174417 as a degree in medical studies by one of the authors (MH) with restricted access.

## Results

### Study selection

**Figure 1:**
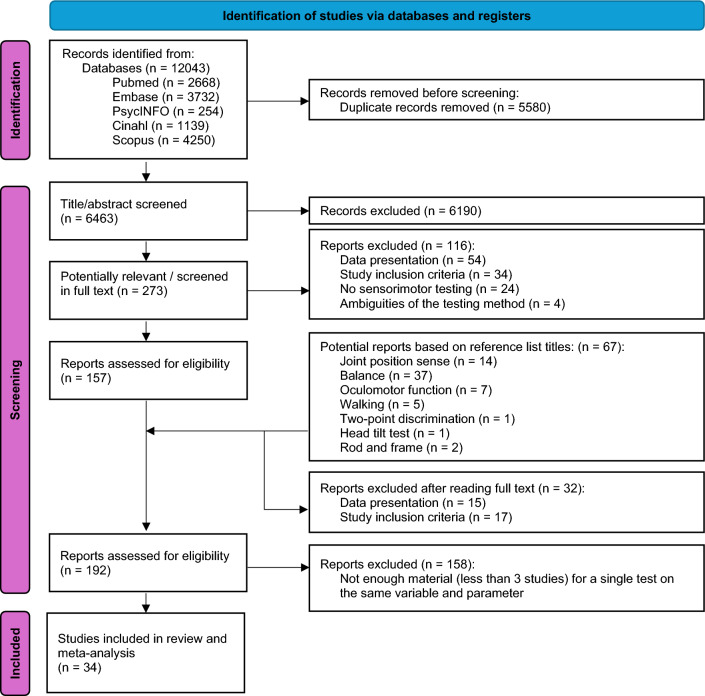
Prisma flow chart demonstrating the screening process.

The literature search retrieved 12,043 studies, 5580 of which were duplicates. Figure 1 details the screening process. The final review included 34 studies^11,12,24–55^.

In the first phase, Cohen’s kappa (95% CI) for inter-rater reliability was 0.57 (0.51–0.62), and inter-rater agreement was 96.9% between the evaluators. In the second phase, Cohen’s kappa was 0.39 (95% CI 0.28–0.50), and inter-rater agreement was 71.1%. In both phases, there was a significant difference (p < 0.05) in the inter-evaluator disagreement.

### Characteristics of included studies

Of the studies included in the review, 25^12,24–47^ were related to joint position sense. The accuracy of joint position sense was assessed by head repositioning error (HRE), defined as the distance between the target's position and the point indicated by the target. All studies evaluated the head repositioning to neutral after movements in different directions. The result was reported as an absolute error either in one test direction (e.g., rotation to the left) or in one movement plane (e.g., total result of left and right rotations). The Fastrak motion tracker and laser were the most common instruments for assessing HRE.

Four included studies^11,48–50^ assessed oculomotor function with the Smooth Pursuit Neck Torsion test (SPNT). Three studies used electro-oculography, and one used video-oculography to record eye movements while following a moving target in neutral and torsional neck positions. The results were reported as a mean gain (the ratio between the eye movements and of the target) in each test position and as a difference between the gain in natural and the average values in the torsional position (SPNT difference).

Six included studies^38,51–55^ assessed balance using a static force platform, which recorded the postural sway. The results were reported as confidence ellipse areas (CEA). CEA was defined as the area of the 95% bivariate ellipse, entailing approximately 95% of the points of the center of the pressure path. The tests were done with eyes closed and open.

Appendix 2 summarizes the included studies, the subjects' demographic data, the test implementations, and the results. In addition, Appendix 3 presents the detailed results of individual studies and the forest plots demonstrating the meta-analysis of each test.

### Methodological quality

**Figure 2:**
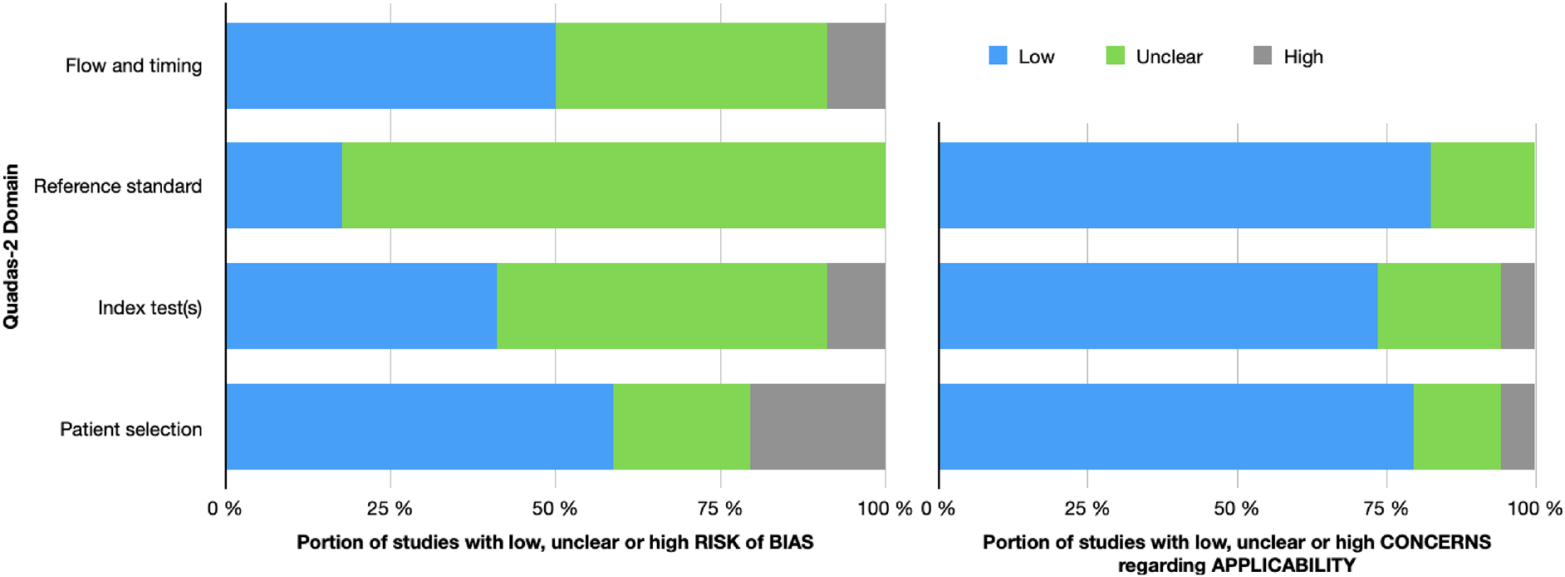
Summary of QUADAS-2 assessment. The portion of studies with low, unclear, or high risk of bias and concerns regarding applicability.

Most included studies provided accurate information on study objective(s), inclusion and exclusion criteria, enlistment, and background characteristics. However, 15 studies (HRE: 12/25 and balance 3/6) had high or unclear risk due to the inclusion and exclusion criteria and enlistment, such as possible selection of subjects. All studies except one accurately described the implementations of the tests, but the reliability of the test used, the accuracy of the measurement tool, or the reference standard of the test often remained unclear. In three studies (all in HRE testing), the testers were not blinded to the group allocation, and in 15 studies (HRE: 12/25, balance: 1/6 and oculomotor function 2/4), the blinding of the testers remained unclear. Half of the studies had a low risk of bias in the flow and timing domain. The test interval, the reporting of those who dropped out, and whether all study subjects were tested remained unclear in 14 studies (HRE: 10/25, balance 2/6 and oculomotor function 2/4). Furthermore, in three studies (all in HRE testing), the risk of bias was high due to the test interval of the study groups and the analysis falling short of the sample size calculations. A summary of the evaluation of the methodological quality of the included studies is shown in Fig. 2 and detailed in Appendix 4.

### Joint position sense

The types of neck pain examined in the studies were non-specific neck pain (NSNP), traumatic neck pain (TNP), and neck pain with dizziness (NPD). However, in the TNP group, two studies^12,31^ divided subjects into dizziness and non-dizziness subgroups, and two studies^27,28^ divided the subjects according to pain severity. Therefore, four different analyses were performed in which the data from these four studies were pooled with other data from TNP studies as follows: TNP with dizziness and mild pain (TNPDM); (2) TNP with dizziness and severe pain (TNPDS); (3) TNP with non-dizziness and mild pain (TNPNDM); and (4) TNP with non-dizziness and severe pain (TNPNDS). The NPD group consisted of individuals with traumatic^12,31^ and non-specific^43^ neck pain.

The sample size in the NSNP group was 568 (range 8–68), in the TNP group 533 (range 18–80), in the NPD group 219 (range 50–93), and in the healthy controls (HC), 908 (range 14–98). Most of the subjects were women (65%). One study^31^ did not report numbers for men and women separately. There was a significant statistical difference in sex distribution between the groups.

The mean age was 39.3 (SD 12.7) in the NSNP group, 39.1 (SD 5.5) in the TNP group, 39.3 (SD 3.9) in the NPD group, and 36.3 (SD 10.8) in HCs. One study^33^ did not report the average age of the subjects, and in one study^25^, the standard deviation value was unclear. Therefore, we excluded these results from the age analysis. Meta-analysis showed that the subjects in the neck pain groups and TNP group were older than those in the HC groups and NSNP group, respectively.

Meta-analysis showed a larger head repositioning error to neutral after cervical rotation in individuals with TNP compared to those with NSNP. However, the difference between the groups was small (ES 0.24). Repositioning error was also larger in all neck pain groups compared to HCs. The extent of the difference (ES 0.4–1.24) between the TNP group and HCs depended on the movement direction tested and the TNP subcategories analyzed. The difference was from medium to large (ES 0.56–0.95) between the NSNP group and HCs and large (1.05–1.49) between the NPD group and HCs in all directions of movement. A summary of the demographic factors and the detailed results of the meta-analysis of the HRE tests between different groups in each direction are shown in Table 1.

**Table 1:** The differences in head reposition error tests between different groups.

Group	Number of studies	n male/female (range)	Difference in sex distribution	Average age (range)	Age difference, ES	Rot/Rot R/Rot L, ES	Flex-Ext/Flex/Ext, ES
TNP	5	48/117 (0–22/11–35)	p < 0.01	43.1 (33.4–49)	1.38	0.24/–/–	
NSNP		47/119 (0–19/9–38)		41.6 (30–49)			
HC	11	113/214 (0–29/11–33)		37.3 (26.9–50)			
TNPDM		120/268 (0–24/11–56)	p = 0.05	39.2 (33.4–49)	0.72	–/0.94/0.94	–/–/0.44
TNPDS		115/270 (0–24/11–56)	p = 0.05	39.8 (33.4–49)	0.77	–/1.24/1.06	–/–/0.57
TNPNDM		105/233 (0–24/11–56)	p = 0.05	39.2 (33.4–49)	0.74	–/0.79/0.85	–/–/0.4
TNPNDS		100/235 (0–24/11–56)	p = 0.05	39.8 (33.4–49)	0.78	–/1.10/0.97	–/–/0.58
HC	18	250/332 (0–61/0–30)	p = 0.01	35.9 (20.1–69.6)	0.55	0.71/0.86/0.95	0.56/0.72/0.59
NSNP		179/373 (0–19/0–49)		39.3 (21–73.2)			
HC	3	63/79 (15–48/29–50)	p < 0.01	36.5 (29.5–46)	0.31	–/1.42/1.49	–/–/1.05
NPD		64/105 (22–42/51–54)		39.3 (35.5–43.3)			

ES: effect size; Ext: extension; n: a number of subjects; Flex-ext: flexion–extension; Flex: flexion; HC: healthy controls; NPD: neck pain with dizziness; NSNP: non-specific neck pain; TNP: traumatic neck pain; Rot: rotation; Rot L: rotation to the left; Rot R: rotation to the right; TNPDM: TNP with dizziness and mild pain; TNPDS: TNP with dizziness and severe pain; TNPNDM: TNP with non-dizziness and mild pain; TNPNDS: TNP with non-dizziness and severe pain.

### Oculomotor function

Two^11,49^ of the four studies divided subjects with TNP into dizziness and non-dizziness subcategories. Therefore, two different analyses were performed in which the data from these two studies were pooled with other data from TNP studies as follows: (1) TNP with dizziness (TNPD), and (2) TNP without dizziness (TNPND).

The sample size in the TNP group was 238 (range 25–50) and 126 (range 23–50) in the HC group. Most of the subjects were women (62%). There was no statistical difference in sex ratios between TNPD and HC groups or TNPND and HC groups, p = 0.55 and p = 0.53, respectively. The mean age was 37.8 (SD 2.2) in the TNPD group, 36.5 (SD 2.8) in the TNPND group and 35.4 (SD 8.0) in HCs.

Meta-analysis showed that the neutral gain was lower, and the SPNT difference was greater in individuals with TNP compared to HCs. The difference was at least moderate (0.53). A summary of the demographic factors and detailed results of the meta-analysis of the SPNT tests between different groups are shown in Table 2.

**Table 2:** Differences in Smooth Pursuit Neck Torsion test between individuals with traumatic neck pain and healthy controls.

Group comparison	Number of studies	n male/female (range)	Average age (range)	Neutral gain, ES	SPNT difference, ES
HC	4	54/72 (7–20/11/30)	35.4 (29.9–47)		
TNPD		64/99 (10–23/14–38)	37.8 (35.5–40.3)	− 0.60	0.82
TNPND		53/85 (8–23/14/38)	36.5 (34–40.3)	− 0.53	0.61

ES: effect size; HC: healthy controls; n: a number of subjects; TNPD: traumatic neck pain with dizziness; TNPND: traumatic neck pain without dizziness.

### Balance

The sample size in the NSNP group was 183 (range 30–85), in the TNP group 54 (range 9–35), and in the HC group 220 (range 10–109). Almost all subjects were women (92%). One study^54^ did not report numbers for women and men separately. There was no statistical difference in sex ratios between the NSNP and HC groups or the TNP and HC groups. The mean age was 39.9 (SD 4.5) in the NSNP group, 43.0 (SD 4.8) in the TNP group, and 37.1 (SD 5.9) in HC. Meta-analysis showed that the subjects in the TNP group were older than those in the HC group.

Meta-analysis showed that the postural sway was larger in individuals with neck pain compared to HCs when the test was done with eyes closed. The difference was small (ES 0.37) between the NSNP group with eyes closed and HCs and large (ES 1.17) between the TNP and HC groups. However, when the test was done with eyes open, there were no differences between the NSNP and HC groups. A summary of the demographic factors and detailed results of the meta-analysis of the balance tests between different groups are shown in Table 3.

**Table 3:** Differences in balance test between different groups.

Group	Number of studies	n male/female (range)	Difference in sex distribution	Average age (range)	Age difference, ES	95% Confidence ellipse area difference, ES
HC	3	3/53 (0–3/10–30)	p = 1.0	35.78 (30.45–41)	4.72	1.17
TNP		3/51 (0–3/6–35)		42.9 (37.7–47)		
HC	3	0/139 (0–0/30–109)	p = 1.0	37.27 (30.45–45)	–	0.37
NSNPEC		0/123 (0–0/38–85)		41.5 (38–45)		
HC	3	12/127 (0–12/18–109)	p = 0.53	39.16 (33.37–45)	–	0.01
NSNPEO		13/102 (0–13/17–85)		40.84 (36.67–45)		

ES: effect size; HC: healthy controls; n: a number of subjects; NSNPEC: non-specific neck pain with eyes closed; NSNPEO: non-specific neck pain with eyes open; TNP: traumatic neck pain.

## Discussion

Our study aimed to clarify sensorimotor control in different types of neck pain and identify possible factors that can affect sensorimotor control test results. We were able to perform a meta-analysis of three commonly used sensorimotor tests: the HRE, SPNT, and balance tests. Our review showed that individuals with neck pain had poorer joint position sense, oculomotor function, and wider postural sway compared to healthy controls. Furthermore, the joint position sense in individuals with TNP was poorer compared to those with NSNP.

### Joint position sense

A head repositioning error of 4.5° has been considered a specific and sensitive cutoff value for distinguishing individuals with neck pain (HRE > 4.5°) from healthy controls (HRE < 4.5°)^23,56^. In our review, the mean repositioning errors were in HCs within the cut-off value in 96% of the studies. On the other hand, in individuals with NSNP and TNP, the mean errors were outside the cut-off value in only 50% and 46% of the studies, respectively. This suggests that some of the results of neck pain groups included in our meta-analysis can be classified as normal. Despite this, individuals with NSNP or TNP showed larger errors compared to HC in the head repositioning to neutral after active cervical rotation (HRE R), extension (HRE E), and flexion (HRE F).

However, our analysis also identified differences in the ability of the HRE tests to distinguish neck pain groups from HCs. In contrast to the HRE R and F tests, where confidence intervals were wide and heterogeneity high, group differences can be considered real for the HRE E test due to narrow confidence intervals, high overall effects (Z-test), and moderate heterogeneity (I^2^). Therefore, our results support using the HRE E test to assess joint position sense in individuals with NSPN and TNP. In addition, our results suggest that the HRE R and F tests are not necessarily clinically accurate enough to identify the differences between individuals with neck pain and healthy individuals.

### Oculomotor function and balance

Individuals with TNP showed lower neutral gain and larger postural sway compared to HC. Although overall effects were high and heterogeneities were low to moderate, confidence intervals were wide, suggesting that group differences between individuals with TNP and HC may not be clinically significant.

There were no differences in postural sway between individuals with NSNP and HCs. Due to insufficient data, we could not perform a meta-analysis of oculomotor function between individuals with NSNP and HCs. Furthermore, we could not compare the mean results and the cut-off values ​​of the SPNT and balance tests because, to our knowledge, no comparable cut-off values ​​have been defined.

### Factors affecting the results

Our review found differences in cervical position sense, oculomotor function and postural balance in individuals with neck pain compared to healthy controls. It has been suggested that factors that cause noise or delay in sensory feedback, such as pain and muscle function changes, may limit our ability to perceive accurately and act precisely^1–3^. However, our results also showed large between-study variations. This may indicate that neck pain itself is not the cause of changes in sensorimotor control. On the other hand, other factors may also be behind the variations in results, such as the subjects’ different symptom profiles and demographic characteristics and the methodological aspects of the studies.

In our review, the results of the subjects who experienced dizziness were reported separately in the five studies, three related to the HRE test^12,31,43^ and two to the SPNT test^11,49^. Our meta-analysis revealed a large group difference between individuals with neck pain and dizziness and HCs in the HRE tests. Furthermore, the difference between the TNP and HC groups seemed to increase in the HRE and SPNT tests when individuals with dizziness were included in the analysis compared to those without dizziness. In the HRE tests, pain intensity also seemed to affect the difference between the TNP group and HCs. Our results were in line with the previous meta-analysis^8^, suggesting that individuals with dizziness and neck pain may have an indication to assess cervical sensorimotor control. It should be noted, however, that we could not perform a separate comparative analysis between the different neck pain groups (= those with and without dizziness or those with mild and severe pain) due to the rule of the three studies we used.

The diversity of the studied groups can also affect the variability of the results, as several studies have reported, for example, in healthy individuals, the effects of age and sex on sensorimotor control^57–59^. According to our meta-analysis, neck pain groups were older than HCs in the HRE and balance tests. However, this analysis may be biased due to the discrepant results in one study^38^. Although De Pauw et al.^38^ reported age variation as a standard deviation, the wide range and large difference between the median and mean raises doubts about the reported indicators. There was also a statistically significant difference between sex ratios in the study groups in the HRE tests but not in the balance test.

Due to the heterogeneous demographic data, we could not perform a more detailed analysis of other factors, such as pain or disability. However, it was interesting to note that studies comparing differences between different types of neck pain in the HRE test reported significantly more pain and disability in the TNP group than in the NSNP group^26,30,38^. Therefore, the small difference observed in our meta-analysis between the TNP and NSNP groups in the HRE test may be due to differences in pain intensity or disability rather than the trauma itself.

We tried to find as much comparable data as possible on sensorimotor tests in patients with neck pain by allowing different study designs in our search. However, this proved to be a challenge. Although our review revealed many sensorimotor tests, there was a large variation in variables, parameters, and analysis methods between studies. For example, the sway amplitude of the posturography was reported in the total envelope area or the 90% or 95% confidence ellipse area. We excluded the studies if we could not collect data from at least three studies that used the same analysis method, parameter, and variable in a single test. Therefore, our review included only three tests, and the variables to be investigated remained small.

However, we pooled the results between the studies even though the tests used different equipment and were carried out slightly differently. For example, in the HRE tests, studies used six different equipment, and various numbers of repetitions, and head rotation ranges when returning the head to the neutral position. Furthermore, in the SPNT test, studies used two different equipment and different velocities of a sinusoidal stimulus, frequencies, and visual angles. In posturography, the equipment and test implementations were mainly comparable between studies.

However, the variability of test equipment and implementation may affect the interpretation of the results observed in our review. To consider these factors related to the reliability and validity of sensorimotor tests, which have been commonly ignored in previous reviews^4–8^, we used the QUADAS-2 tool to assess the quality of the studies. The quality assessment of the studies revealed the need to develop gold standards for sensorimotor control tests. For example, in the HRE and balance tests, many studies did not consider factors affecting the reliability of the test, such as the number of test repetitions or the test duration^56,60^. Furthermore, some of the studies used equipment with poor or unclear accuracy. For example, the SPNT test mainly used electro-oculography, which does not match the accuracy of the gold standard used for eye movement assessment, the scleral search coil technique, or comparable video-oculography^61–63^. Therefore, our meta-analysis includes tests with varying reliability and validity. This weakens the quality of our review. Despite this, we did not stratify the analysis based on the studies’ risk of bias, as this may lead to selection bias related to the risk assessment tool used^64^.

Our review's screening and synthesis processes may also involve factors that may have influenced the results and conclusions we reached. First, systematic reviews aim to identify and synthesize relevant evidence regardless of geographical provenance or publication language^65^. Since our search included only studies in English, it is possible that studies in languages other than English could have influenced our research results. Second, it should also be noted that the number of studies excluded in both screening phases differed significantly between evaluators. Although this may also indicate a more comprehensive screening of studies, it does not eliminate the possibility that we have missed relevant studies, for example, due to the lack of information in the titles and abstracts of the studies^66^.

## Conclusion

The sensorimotor tests included in our review are currently widely used for neck pain. Our results support using sensorimotor tests in patients with neck pain and its associated disorders. However, our results also suggest that the background of the cervical sensorimotor impairment is not necessarily neck pain or trauma alone but rather a combination of different factors. Dizziness and severe pain seem to play a role in discriminating individuals between those with neck pain and those without symptoms. On the other hand, our results should be treated with caution, as the quality and performance of the measurement tools used in the studies included in the review can be questioned. In future studies, it might be useful to differentiate patients with neck pain into subgroups based on the symptom and demographic profiles to assess other factors that significantly affect cervical sensorimotor control. Furthermore, future studies should also focus on assessing the clinimetric properties of the cervical sensorimotor control tests.

### Supplementary Information

Supplementary Information 1.

Supplementary Information 2.

Supplementary Information 3.

## Data Availability

Data are available from the corresponding author upon reasonable request.
