# Supplementary material for: Sensorimotor tests in patients with neck pain and its associated disorders: a systematic review and meta-analysis
Source: Sci Rep. 2024 Jun 4;14:12764. doi: 10.1038/s41598-024-63545-3 (PMC11150393; doi:10.1038/s41598-024-63545-3)
Supplement: Supplementary file 1 — Supplementary Information 1. [file 41598_2024_63545_MOESM1_ESM.pdf]

Supplementary Information

Title: Sensorimotor tests in patients with neck pain and its associated disorders: A systematic review and meta-analysis

Authors: Niklas Särkilahti, Mhc[a,b], Milka Hirvonen, BM[c], Joona Lavapuro, BM[c], Jani Takatalo, PhD MD[d,e], Eliisa Löyttyniemi, MSc[f], Olli Tenovuo, Prof. PhD MD[a,g]

[a] University of Turku, Faculty of Medicine, Dept. of Clinical Neurosciences, Turku, Finland.

[b] Neurocenter, Turku University Hospital, Turku, Finland.

[c] University of Turku, Faculty of Medicine, Turku, Finland.

[d] Medical Research Center Oulu, University of Oulu, Finland.

[e] Loisto Terveys, Oulu, Finland.

[f] University of Turku and Turku University Hospital, The Department of Biostatistics, Turku, Finland.

[g] Turku Brain Injury Centre, Turku University Hospital, Turku, Finland.

Appendix 1. Full search strategy

Pubmed:

(neck*[Tiab] OR cervic*[Tiab] OR whiplash*[Tiab])

AND

(Proprioception[Mesh] OR ”Eye Movements”[Mesh] OR sensorimotor[Tiab] OR ”sensory motor”[Tiab] OR ”sensory receptor”[Tiab] OR ”sensory feedback”[Tiab] OR propriocept*[Tiab] OR vestibular[Tiab] OR ”postural control”[Tiab] OR ”postural sway”[Tiab] OR balance[Tiab] OR mechanorecept*[Tiab] OR ”motor control”[Tiab] OR kinesthetic[Tiab] OR kinaesthesia[Tiab] OR kinematics[Tiab] OR ”joint position”[Tiab] OR ”joint reposition”[Tiab] OR ”joint movement”[Tiab] OR ”position sense”[Tiab] OR ”head reposition”[Tiab] OR “head position”[Tiab] OR ”eye movement”[Tiab] OR oculomotor[Tiab] OR ”eye-head coordination”[Tiab])

AND

(”Sensitivity and Specificity”[Mesh] OR ”Reproducibility of Results”[Mesh] OR ”Odds ratio”[Mesh] OR ”sensitivity”[Tiab] OR ”specificity”[Tiab] OR ”reliability”[Tiab] OR ”validity”[Tiab] OR ”reproducibility”[Tiab] OR ”repeatability”[Tiab] OR ”accuracy”[Tiab] OR ”detection”[Tiab] OR ”acuity”[Tiab] OR ”discrimination”[Tiab] OR ”difference”[Tiab] OR ”coefficient”[Tiab] OR ”intra-examiner”[Tiab] OR ”intraexaminer”[Tiab] OR ”inter-examiner”[Tiab] OR ”interexaminer”[Tiab] OR ”intra-observer”[Tiab] OR ”intraobserver”[Tiab] OR ”inter-observer”[Tiab] OR ”interobserver”[Tiab] OR “odds ratio”[Tiab])

EMBASE:

(neck*:ab,ti OR cervic*:ab,ti OR whiplash*:ab,ti)

AND

('sensorimotor function'/exp OR 'Proprioception'/exp OR ’motor control'/exp OR ’head position'/exp OR ’eye movement'/exp OR sensorimotor:ab,ti OR 'sensory motor':ab,ti OR 'sensory receptor':ab,ti OR 'sensory feedback':ab,ti OR propriocept*:ab,ti OR vestibular:ab,ti OR 'postural control':ab,ti OR 'postural sway':ab,ti OR balance:ab,ti OR mechanorecept*:ab,ti OR 'motor control':ab,ti OR kinesthetic:ab,ti OR kinaesthesia:ab,ti OR kinematics:ab,ti OR 'joint position':ab,ti OR 'joint reposition':ab,ti OR 'joint movement':ab,ti OR 'position sense':ab,ti OR 'head reposition':ab,ti OR 'head position':ab,ti OR 'eye movement':ab,ti OR oculomotor:ab,ti OR 'eye-head coordination':ab,ti)

AND

('reliability'/exp OR 'accuracy'/exp OR sensitivity:ab,ti OR specificity:ab,ti OR reliability:ab,ti OR validity:ab,ti OR reproducibility:ab,ti OR repeatability:ab,ti OR accuracy:ab,ti OR detection:ab,ti OR acuity:ab,ti OR discrimination:ab,ti OR difference:ab,ti OR coefficient:ab,ti OR 'intra-examiner':ab,ti OR intraexaminer:ab,ti OR 'inter-examiner':ab,ti OR interexaminer:ab,ti OR 'intra-observer':ab,ti OR intraobserver:ab,ti OR 'inter-observer':ab,ti OR interobserver:ab,ti OR 'odds ratio':ab,ti)

PSYCINFO:

(TI neck* OR AB neck* OR TI cervic* OR AB cervic* OR TI whiplash* OR AB whiplash*)

AND

(DE "Proprioception" OR DE "Eye Movements" OR TI sensorimotor* OR AB sensorimotor* OR TI ”sensory motor” OR AB ”sensory motor” OR TI ”sensory receptor” OR AB ”sensory receptor” OR TI ”sensory feedback” OR AB ”sensory feedback” OR TI propriocept* OR AB propriocept* OR TI vestibular OR AB vestibular OR TI ”postural control” OR AB “postural control” OR TI ”postural sway” OR AB ”postural sway” OR TI balance OR AB balance OR TI mechanorecept*OR AB mechanorecept* OR TI ”motor control” OR AB “motor control” OR TI kinesthetic OR AB kinesthetic OR TI kinaesthesia OR AB kinaesthesia OR TI kinematics OR AB kinematics OR TI ”joint position” OR AB ”joint position” OR TI ”joint reposition” OR AB “joint reposition” OR TI ”joint movement” OR AB “joint movement” OR TI ”position sense” OR AB “position sense” OR TI ”head reposition” OR AB “head reposition” OR TI “head position” OR AB “head position” OR TI ”eye movement” OR AB “eye movement” OR TI oculomotor OR AB oculomotor OR TI ”eye-head coordination” OR AB “eye-head coordination”)

AND

(TI ”sensitivity” OR AB “sensitivity” OR TI ”specificity” OR AB “specificity” OR TI ”reliability” OR AB ”reliability” OR TI ”validity” OR AB ”validity” OR TI ”reproducibility” OR AB ”reproducibility” OR TI ”repeatability” OR AB ”repeatability” OR TI ”accuracy” OR AB ”accuracy” OR TI ”detection” OR AB ”detection” OR TI ”acuity” OR AB ”acuity” OR TI ”discrimination” OR AB ”discrimination” OR TI ”difference” OR AB ”difference” OR TI ”coefficient” OR AB “coefficient” OR TI ”intra-examiner” OR AB ”intra-examiner” OR TI ”intraexaminer” OR AB ”intraexaminer” OR TI ”inter-examiner” OR AB ”inter-examiner” OR TI ”interexaminer” OR AB ”interexaminer” OR TI ”intra-observer” OR AB ”intra-observer” OR TI ”intraobserver” OR AB ”intraobserver” OR TI ”inter-observer” OR AB ”inter-observer” OR TI ”interobserver” OR AB ”interobserver” OR TI “odds ratio” OR AB “odds-ratio”)

**CINAHL:**
(TI neck* OR AB neck* OR TI cervic* OR AB cervic* OR TI whiplash* OR AB whiplash*)
AND
((MH ”Proprioception") OR (MH "Motor Skills") OR (MH "Kinematics") OR (MH "Eye Movements") OR TI sensorimotor* OR AB sensorimotor* OR TI ”sensory motor” OR AB ”sensory motor” OR TI ”sensory receptor” OR AB ”sensory receptor” OR TI ”sensory feedback” OR AB ”sensory feedback” OR TI propriocept* OR AB propriocept* OR TI vestibular OR AB vestibular OR TI ”postural control” OR AB “postural control” OR TI ”postural sway” OR AB ”postural sway” OR TI balance OR AB balance OR TI mechanorecept*OR AB mechanorecept* OR TI ”motor control” OR AB “motor control” OR TI kinesthetic OR AB kinesthetic OR TI kinaesthesia OR AB kinaesthesia OR TI kinematics OR AB kinematics OR TI ”joint position” OR AB ”joint position” OR TI ”joint reposition” OR AB “joint reposition” OR TI ”joint movement” OR AB “joint movement” OR TI ”position sense” OR AB “position sense” OR TI ”head reposition” OR AB “head reposition” OR TI “head position” OR AB “head position” OR TI ”eye movement” OR AB “eye movement” OR TI oculomotor OR AB oculomotor OR TI ”eye-head coordination” OR AB “eye-head coordination”)
AND
((MH "Repeated Measures") OR (MH "Kappa Statistic") OR TI ”sensitivity” OR AB “sensitivity” OR TI ”specificity” OR AB “specificity” OR TI ”reliability” OR AB ”reliability” OR TI ”validity” OR AB ”validity” OR TI ”reproducibility” OR AB ”reproducibility” OR TI ”repeatability” OR AB ”repeatability” OR TI ”accuracy” OR AB ”accuracy” OR TI ”detection” OR AB ”detection” OR TI ”acuity” OR AB ”acuity” OR TI ”discrimination” OR AB ”discrimination” OR TI ”difference” OR AB ”difference” OR TI ”coefficient” OR AB “coefficient” OR TI ”intra-examiner” OR AB ”intra-examiner” OR TI ”intraexaminer” OR AB ”intraexaminer” OR TI ”inter-examiner” OR AB ”inter-examiner” OR TI ”interexaminer” OR AB ”interexaminer” OR TI ”intra-observer” OR AB ”intra-observer” OR TI ”intraobserver” OR AB ”intraobserver” OR TI ”inter-observer” OR AB ”inter-observer” OR TI ”interobserver” OR AB ”interobserver” OR TI “odds ratio” OR AB “odds-ratio”)

**SCOPUS:**
TITLE-ABS (neck* OR cervic* OR whiplash*)
AND
TITLE-ABS (sensorimotor OR "sensory motor" OR "sensory receptor" OR "sensory feedback" OR propriocept* OR vestibular OR "postural control" OR "postural sway" OR balance OR mechanorecept* OR "motor control" OR kinesthetic OR kinaesthesia OR kinematics OR "joint position" OR "joint reposition" OR "joint movement" OR "position sense" OR "head reposition" OR "head position" OR "eye movement" OR oculomotor OR "eye-head coordination")
AND
TITLE-ABS ("sensitivity" OR "specificity" OR "reliability" OR "validity" OR "reproducibility" OR "repeatability" OR "accuracy" OR "detection" OR "acuity" OR "discrimination" OR "difference" OR "coefficient" OR "intra-examiner" OR "intraexaminer" OR "inter-examiner" OR "interexaminer" OR "intra-observer" OR "intraobserver" OR "inter-observer" OR "interobserver" OR "odds ratio")

Appendix 2. Summary of the included studies, the subjects' demographic data, the test implementations, and the results.

Citation	Study design	Subjects	Duration of symptoms	Description of test	Instrument	Summary of results
**Joint position sense**						
**Alalawi et al. 2022 (44)**	A cross-sectional observational study, followed by a longitudinal analysis	Total n=45; RNSNP n=22 (m36%/f64%), mean (SD) age 31.0(11.8); NSNP n=8 (m22%/f88%), mean (SD) age 33.6(8.7); C n=15 (m40%/f60%), mean (SD) age 31.1(5.7)	Chronic (≥3 months)	Sitting on the chair. Movement directions: 1) Rotation to the right, 2) rotation to the left. 3 repetitions.	Wearable IMU	Mean (SD) °: NSNP 1) 5.5(5.9), and 2) 5.2(5.2); C 1) 3.8(2.1), and 2) 4.2(2.8).
**Alalawi et al. 2022 (45)**	An observational case-control study	Total n=60; WAD n=18 (m4/f14), mean (SD) age 38.7(12.0; C n=42 (m9/f33)), mean (SD) age 38.4(10.2).	Acute (≤15 days)	Sitting on the chair. Movement directions: 1) rotation to the right and 2) rotation to the left. 3 repetitions	BTS G-WALK® sensor system	Mean (SD) °: WAD 1) 3.4(2.1), and 2) 3.8(2.4); C 1) 3.2(2.1), and 2) 3.1(2.6).
**Cheever et al. 2017 (37)**	A cross-sectional study	Total n=40: NSNP n=22 (m9/f13), mean (SD) age 25.5 (9.75); C n=18 (m9/f9), mean (SD) age 23 (5.91).	NR	Sitting on the chair which was placed 90 cm. from a target. Head movement with maximum range of motion. Movement directions: 1) Rotation to the right, 2) Rotation to the left, 3) Extension, and 4) Flexion. 8 repetitions.	A laser device	Mean (SD) °: WAD 1) 3.70°(1.9), 2) 4.00(2.10), 3) 3.5(1.80), and 4) 3.40(1.60); NSNP 1) 3.70(1.60), 2) 3.60(3.00), 3) 2.90(1.30), and 4) 2.80(1,20); C 1) 3.1(1.3), 2) 3.5(1.3), 3) 2.70(1.00), and 4) 2.90(0.90).
**Cid et al. 2022 (46)**	An intra-rater reliability study	Total n=41; NSNP f n=13, mean (SD) age 26.6(4.2); C m n=14, mean (SD) age 24.4(3.0); C f n=14, mean (SD) age 23.1(3.4).	Chronic (≥3 months)	Sitting on the chair which was placed 90 cm. from a target. Head movement with maximum but comfortable limits. Movement directions: 1) Rotation to the right, and 2) Rotation to the left. 10 repetitions.	A laser device	Mean (SD) °: NSNP 1) 3.03(0.9), and 2) 3.67(1.8); C m 1) 3.15(1.0), and 2) 3.03(0.6); C f 1) 2.88(0.6), and 2) 3.03(0.7).
**Dugailly et al. 2015 (36)**	A reliability and validity study	Total n=71; NSNP n=35 (m11/f24), mean (SD) age 42(8); NSNP n=35 (m11/f24), mean (SD) age 42(8); C n=36 (m14/f22), mean (SD) age 42(5).	Chronic (>6 months)	Sitting on the chair which was placed 180 cm. from a target. Head movement with maximum range of motion. Test 1 = Slow movement, Test 2 = Fast movement. Movement directions: 1) Rotation to the right, 2) Rotation to the left, 3) Extension, and 4) Flexion. 6 repetitions.	A laser device	Mean (SD) °: Test 1 NSNP 1) 5.3(2.5), 2) 5.5(2.7), 3) Ext 7.3(3.4), and 4) 5.1(2.6); Test 2 NSNP 1) 5.3(2.3), 2) 5.3(3), 3) Ext 7.6(3.5), and 4) 5.6(2.8); Test 1 C 1) 3(1.2), 2) 3(1.4), 3) Ext 3.5(1.3), and 4) 3.1(1.7); Test 2 C 1) 3.3(1.3), 2) 3.3(1.7), 3) 4(2.1), and 4) 3.5(2.8).
**Elsig et al. 2014 (35)**	A case-control study	Total n=60; NSNP n=30 (m5/f25), mean (SD) age 36.9(13.62); C n=30 (m5; f25), mean (SD) age 37.2(13.5).	Chronic (>6 months)	Position and movement range NR. Movement directions: 1) Rotation to the right, 2) Rotation to the left, 3) Extension, and 4) Flexion. 8 repetitions.	A laser device	Mean (SD) °: NSNP 1) 3.27(1.72), 2) 3.1(1.15), 3) Ext 3.19(1.31), and 4) 3.43(1.75); C 1) 2.78(0.87), 2) 2.58(0.83), 3) 2.65(0.95), and 4) 1.75(0.98).
**Ghamkhar et al. 2020 (41)**	A cross-sectional study	Total n=60; NSNP n=30 (m8/f22), mean (SD) age 35.35(12.6); C n=30 (m10/f20), mean (SD) age 28.72(8.51).	Chronic (≥6 months)	Sitting on the chair which was placed 90 cm. from a target. Head movement with maximum comfortable range. Movement directions: 1) Rotation to the right, 2) rotation to the left, 3) extension, 4) flexion. 4 repetitions.	A laser device	Mean (SD) °: NSNP 1) 10.99(5.53), 2) 11.95(4.98), 3) 11.24(4.50), and 4) 9.15(3.59); C 1) 9.82(3.68), 2) 9.06(3.51), 3) 10.82(3.27), and 4) 8.65(3.14)
**Goncalves et al. 2019 (40)**	A reliability and validity study	Total n= 66; NSNP n=33 (m7/f26), mean (SD) age 43.6(13.3); C n=33 (m7/f26), mean (SD) age 43.5(14.1).	Chronic (>3 months)	Sitting on the chair which was placed 90 cm. from a target. Head movement with maximum range of motion. Movement directions: 1) Rotation to the right, and 2) Rotation to the left. 6 repetitions.	A laser device	Mean (SD) °: NSNP 1) 5.12(2.67), and 2) 5.01(3.25); C 1) 3.79(1.71), and 2) 3.87(2.1).
**Grip et al. 2007 (29)**	NR	Total n=67: WAD n=22 (m5/f17), mean (SD) age 49(15); NSNP n=21; (m7/f14), mean (SD) age 49(16); C n=24; (m8/f16), mean (SD) age 50(18).	Chronic (>3 months)	Sitting on the chair which was placed 100cm. from target. Head movement from target: 1) from 30° right rotation, 2) from 30° left rotation, 3) from 25° extension, and 4) from 25° flexion to neutral. 5 repetitions.	Proreflex system	Mean (SD) °: WAD 1) 3.7(1.9), 2) 4.0(2.1), and 3) 3.5(1.8), and 4) 3.4(1.6); NSNP 1) 3.7(1.6), 2) 3.6(3.0), 3) 2.9(1.3), and 2.8(1.2); C 1) 3.1(1.3), 2) 3.5(1.3), 3) 2.7(1.0), and 4) 2.9(0.9).
**Heikkilä et al. 1998 (25)**	A 2-year review of consecutive patients admitted to the emergency unit after whiplash injury.	Total n=66: WAD n=27 (m14/f13), mean age 33.8; C n=39 (m15/f24), mean age 35.	Chronic (6 months - 10 years)	Sitting on the chair which was placed 90 cm. from a target. Head movement with maximum range of motion. Movement directions: 1) Rotation to the right, 2) Rotation to the left, 3) extension, and 4) Flexion. 10 repetitions.	A laser device	Mean (SD) °: WAD 1) 4.32(2.86), 2) 3.99(3), 3) 5.21(3.46), and 4) 5.12(3.6); C 1) 2.78(2), 2) 2.69(1.78), 3) 2.84(1.84), and 4) 2.54(2.13).
**Hill et al. 2009 (31)**	A comparative study of error calculation	Total n=150: WADD n=50, mean (SD) age 35.5(8.1); WADND n=50, mean (SD) age 35(1.9); C n=50, mean (SD) age 29.5(8.3).	Chronic (>3 months)	Sitting on the chair. Head movement within comfortable limits. Movement directions: 1) Rotation to the right, 2) Rotation to the left, and 3) Extension. 3 repetitions.	Fastrak	Mean (SE) °: WADD 1) 4.55(0.4), 2) 4.01(0.3), and 3) 3.61(0.3); WADND 1) 2.93(0.4), 2) 3.07(0.3), and 3) 2.84(0.3); C 1) 3.16(0.4), 2) 2.47(0.4), and 3) 3.01(0.3).
**Kristjansson et al. 2003 (26)**	A case–control study	Total n=63; WAD n=22 (m11/f11), mean (SD) age 33.4(10.6); NSNP n=20 (m11/f9), mean (SD) age 30.0 (9.1); C n=21 (m10/f11), mean (SD) age 26.9(6.4).	Chronic (3–48 months)	Sitting on the chair. Head movement within comfortable limits. Movement directions: Rotation. 3 repetitions.	Fastrak	Mean (SD) °: NSNP 3.33(1.42); WAD 4.14(1.58); C 2.48(1.12).

**Lopez-de-Uralde-Villanueva et al. 2020 (42)**	A cross-sectional, observational, descriptive and nonprobabilistic design	Total n=183: NSNP n=68 (m19/f49), mean (SD) age 39.91(14.36); C n=48; (m18/f30); mean (SD) age 26.6(14.14)	Chronic (>3 months)	Sitting on the chair which was placed 90 cm. from a target. Head movement in a sub-maximal manner. Movement directions: 1) Rotation, 2) Extension, and 3) Flexion. 3 repetitions	A laser device	Mean (SD) °: NSNP 1) 4.54(2.59), 2) 5.21(3.78), and 3) 4.10(2.34); C 1) 3.89(1.49), 2) 4.24(2.37), and 3) 3.97(1.50).
**Micarelli et al. 2020 (43)**	NR	Total n=191; CD n=93 (m42/f51), mean (SD) age 43.6(13.3); C n=98 (m48/f50), mean (SD) age 43.5(14.1).	Chronic (>3 months)	Sitting on the chair which was placed 90 cm. from a target. Movement directions: 1) Rotation to the right, 2) Rotation to the left, 3) Extension, and 4) Flexion. 3 repetitions.	A laser device	Mean (SD) °: CD 1) 5.32(1.26), 2) 5.05(1.04), 3) 4.97(1.23), and 4) 4.96(1.19); C 1) 2.43(0.66), 2) 2.48(0.61), 3) 2.63(0.62), and 4) 2.59(0.6).
**Moustafa et al. 2022 (47)**	A cross-sectional, case control design	Total n=90; WAD n=30 (m10/f20), mean (SD) age 48(2); NSNP n=30 (m10/f20), mean (SD) age 47(1) ; C n=30 (m10/f20), mean (SD) age 48(2).	Chronic (≥3 months - 12 months)	Sitting on the chair; Head movement from 30°. Movement directions: 1) Rotation to the right, and 2) Rotation to the left. 3 repetitions.	NR	Mean (SD) °: WAD 1) 3.1(0.611), and 2) 3.03(0.61); NSNP 1) 2.37(0.44), and 2) 2.37(0.44); C 1) 0.35(0.20), and 2) 0.35(0.20).
**Van den Oord et al. 2010 (33)**	NR	Total n=117; NSNP n=83 (m83/f0), age NR; C n=34 (m34/f0), age NR	NR	Sitting on the chair. Head movement: in a sub-maximal manner. Movement directions: 1) Rotation, and 2) Flexio-extension. 10 repetitions.	Zebris	Mean (SD) °: NSNP pilot 1) 1.9(0.6), and 2) 2.8(1); NSNP aircrew 1) 2.1(0.8), and 2) 3.3(1.4); C pilot 1) 1.8(0.6), and 2) 3.1(1.2); C aircrew 1) 2.1(0.7), and 2) 3(0.8).
**De Pauw et al. 2018 (38)**	A case-control study	Total n=103: WAD n=35 (m0/f35), mean (SD) age 47(1.11); NSNP n=38 (m0/f38), mean (SD) age 38(1.41); C n=30 (m0/f30), mean (SD) age 30.45(1.15).	Chronic (>3 months)	Sitting on the chair which was placed 90 cm. from a target. Head movement with maximum range of motion. Movement directions: 1) Rotation, and 2) Flexio-extension. 10 repetitions.	A laser device	Mean (SD) °: WAD 1) 4.3(2.16), and 2) 3.97(2.05); NSNP 1) 3.81(1.47), and 2) 3.5(1.2); C 1) 3.46(1.44), and 2) 3.04(0.99).
**Portelli et al. 2018 (39)**	A 2-Group Comparative Observational Study	Total n=44: NSNP n=22 (m9/f13), mean (SD) age 21.0(3.5); C n=22 (m7/f15), mean (SD) age 20.1(1.2).	Chronic (>3 months)	Sitting on the chair which was placed 90 cm. from a target. Head movement with 50% of maximum range of motion. Movement directions: 1) Rotation to the right, 2) Rotation to the left, 3) Extension, and 4) Flexion. 3 repetitions.	A laser device	Mean (SD) °: NSNP 1) 4.27(1.49), 2) 4.48(1.84), 3) Ext 3.98(1.85), and 4) 3.91(1.44); C 1) 3.95(1.34), 2) 3.62(1.57), 3) 3.35(1.46), and 4) 2.95(1.17).
**Revel 1991 (24)**	NR	Total n=60: NSNP n=30 (m19/f20), mean age 45; C n=30 (m10/f20), mean age 44.	Chronic	Sitting on the chair which was placed 90 cm. from a target. Head movement with maximum range of motion. Movement directions: 1) Rotation to the right, 2) Rotation to the left, 3) extension and 4) flexion. 10 repetitions.	A laser device	Mean (SD) °: NSNP 1) 6.1(2,23), 2) 6.11(2,1), 3) 547(2.29), and 4) 5.48(0.23); C 1) 3.5(0.76), 2) 3.5(1.14), 3) 3.43(0.82), and 4) 3.31(1.14).
**Roren et al. 2009 (32)**	A reliability study	Total n=82; NSNP n=41 (m11/f30), mean (SD) age 54.7(14.2), C n=41 (m18/f23), mean (SD) age 30.5 (11.4).	NR	Sitting on the chair which was placed 90 cm. from a target. Head movement with maximum range of motion. Movement directions: Rotation; 10 repetitions.	A laser device	Mean (SD) °: NSNP 6.3(12.4); C 3.6(0,8).
**Sterling et al. 2003 (27)**	A prospective longitudinal design	Total n=86; WADR n=25(m40%/f60%), mean (SD) age 33.5(10.2); WADM n=22 (m36%/f64%), mean (SD) age 34.7(12.6); WADS n=19 (m16%/f84%), mean (SD) age 41.3(13.6); C n=20 (m40%/f60%), mean (SD) age 40.1 (13.6).	Acute (1–3 months)	Sitting on the chair. Head movement within comfortable limits. Movement directions: 1) Rotation to the right, 2) Rotation to the left, and 3) Extension. 3 repetitions.	Fastrak	Mean (SE) °: WADR 1) 3.6(0.3); 2) 3.0(0.2), and 3) 3,3(0.3); WADM 1) 2.7(0.3), 2) 2,7(0.2) and 3) 3.4(0.3); WADS 1) 4.8(0.3), 2) 3.2(0.3), and 3) 4.1(0.3); C 1) 2.7(0.3), 2) 2.6(0.3), and 3) 2.8(0.3).
**Sterling et al. 2004 (28)**	An experimental study	Total n=100: WAD n=80 (m24/f56), mean (SD) age 33.5(14.7); WADM n=36 (m47.1%/f52.9%), mean (SD) 30.1(13.2); WADMod n=32 (m12.9%/f87.1%), mean (SD) 34.1(14.3); WADS n=12 (m27.3%/72.7%), mean (SD) age 39.5(14.8); C n=20 (m9; f11), mean (SD) age 39.5(14.6).	Acute (≤ 1 month)	Position and movement range NR. Movement directions: 1) Rotation to the right, 2) Rotation to the left, and 3) Extension. 3 repetitions.	Fastrak	Mean (SE) °: WADM 1) 2.6(0.3), 2) 2.4(0.2), and 3) 3.6(0.4); WADMod 1) 4.5(0.4), 2) 2.4(0.2), and 3) 3.5(0.5); WADS 1) 4.5(0.7), 2) 3.3(0.5), and 3) 5.4(0.9); C 1) 2.3(0.5), 2) 2.3(0.3), and 3) 2.9(0.6).
**Treleaven et al 2003 (12)**	NR	Total n=146. WADD n=76 (m22/f24), mean (SE) age 39.11(1.8); WADND n=26 (m7/f19), mean (SE) age 40.23(1.9); C n=44 (m15/f29), mean (SE) age 34.1(1.8).	Chronic (>3 months)	Sitting on the chair. Head movement within comfortable limits. Movement directions: 1) Rotation to the right, 2) Rotation to the left, and 3) Extension. 3 repetitions.	Fastrak	Mean (SE) °: WADD 1) 4.5(0.3), 2) 3.9(0.3), and 3) 3.5(0.3); WADND 1) 2.9(0.4), 2) 2.8(0.4), and 3) 3.5(0.4); C 1) 2.5(0.2), 2) 2(0.2), and 3) 2.4(0.3).
**Uthaikhup et al. 2012 (34)**	NR	Total n=40; NSNP n=20 (m8/f12), mean (SD) age 73.2(6.2); C n=20 (m6; f14), mean (SD) age 69.55(4.2).	Chronic (>3 months)	Position and movement range NR. Movement directions: 1) Rotation to the right, 2) Rotation to the left, and 3) Extension. 3 repetitions.	Fastrak	Mean (SD) °: NSNP 1) 5.5(3.1), 2) 5.1(4), and 3) 5.2(3.4); C 1) 4.2(2.2), 2) 2.8(1.8), and 3) 3.6(2.4).
**Woodhouse et al. 2008 (30)**	A case-control study	Total n=173; WAD n=56 (m22/f34), mean (SD age 38.19(10.8); NSNP n=57 (m19/f38), mean (SD) age 43.7(12.6); C n=57 (m29/f28), mean (SD) age 38.2 (10.9).	Chronic (6–10 months)	Sitting on the chair which was placed 150 cm. from a target. Head movement within comfortable limits. Movement directions: Rotation. 2 repetitions.	Fastrak	Mean (SD) °: NSNP 3.17(1.1); WAD 3.35(1.6); C 2.86(1.2).

**Oculomotor function**						
**Dispenza et al. 2011 (50)**	A prospective case–control study	Total n=60: WAD n=37 (m23/f14), mean (range) age 36.5(21-53); C n=23 (m12/f11), mean (range) age 30.4(19.49).	WAD group A 1-2 months; WAD group B 2-6 months; WAD group C 7-12 months	Sitting on the chair at 42 cm from the display. The operator hand-fixed the head. The test was performed in 3 different starting positions: 1) the neck in a neutral position, 2) trunk rotation 30° to the left with neck neutral and 3) trunk rotation 30° to the right with neck neutral. Each trial was of 20 cycles of 57 seconds.	Video-oculography	Mean (SD) smooth pursuit gain: WAD group A 1) 0.85(0.03); WAD group B 1) 0.87(0.04), WAD group C 1) 0.87(0.03); WAD 1) 0.87(0.04), 2) 0.86(0.03), and 3) 0.87(0.04); C 1) 0.87 (0.04).
**Prushansky et al 2004 (48)**	A comparative study	Total n=49: WAD n=26 (m10/f16), mean (SD) age 40.3(10.6); C n=23 (m7/f16), mean (SD) age 34.2(13.7).	Chronic (≥6 months)	Sitting on the chair. The test was performed in 3 different starting positions: 1) the neck in a neutral position, 2) trunk rotation 30° to the left with neck neutral and 3) trunk rotation 30° to the right with neck neutral.	Chartr electoro-oculoghraphy	Mean (SD) smooth pursuit gain: WAD 1) 0.79(0.11), 2) 0.75(0.01), and 3) 0.74(0.10); C 1) 0.86(0.01), 2) 0.80(0.01), and 3) 0.82(0.14)
**Tjell et al. 1998 (11)**	A consecutive, prospective, double-blind clinical study	Total n=105: WADD n=50 m (19/f31), mean (range) age 39(18-60); WADND n=25 (m8/f17), mean (range) age 34(21-63); C n=30 (m15/f15), mean (range) age 47(29-59).	Chronic (≥6 months)	Sitting on the chair. The test was performed in 3 different starting positions: 1) the neck in a neutral position, 2) trunk rotation 45° to the left and right with neck neutral (SPNT diff).	Cadwell electro-oculography	Mean (SD) smooth pursuit gain: WADD 1) 0.83(0.09), 2) 0.14(0.08); WADND 1) 0.85(0.06), 2) 0.10(0.11); C 1) 0.87(0.05), 2) 0.02(0.02).
**Treleaven et al. 2005 (49)**	A prospective, 3-group, observational design	Total n=150: WADD n=50 (m12/f38), mean (range) age 35.5(19-46); WADND n=50 (m12/f38), mean (range) age 35.0(18-46); C n=50 (m20/f30), mean (range) age 29.9(19-45).	Chronic (≥3 months)	Sitting on the chair. The examiner gently held the head still during the testing. The test was performed in 3 different starting positions: 1) the neck in a neutral position, 2) torso 45° to the left with neck neutral and 3) torso turned 45° to the right with neck neutral.	Cleartrace ConMed electrooculography	Mean (SE) smooth pursuit gain: WADD 1) 0.81(0.01), 2) 0.70(0.02), 3) 0.69(0.02), and SPNT 0.11(0.01); WADND 1) 0.82(0.01), 2) 0.74(0.02), 3) 0.78(0.12), and SPNT 0.07(0.01); C 1) 0.88(0.01), 2) 0.87(0.02), 3) 0.88(0.02), and SPNT 0.01(0.01).
**Balance**						
**Alizadeh et al. 2022 (55)**	An observational, cross-sectional study with a mixed factorial design	Total n=60: NSNP n=30 (m13/f17), mean (SD) age 36.67(1.75); C n=30 (m12/f18), mean (SD) age 33.37(1.96).	Chronic (≥6 months)	Barefoot standing with arms hanging by their trunk, foot position was standardised. Balance tasks: 1) standing with eyes open and head in a neutral position, 2) standing on a foam surface with eyes open and head in a neutral position, 3) standing with eyes open and maximum extension of the cervical spine, and 4) standing with eyes closed and maximum extension of the cervical spine—test duration: 45 seconds, 3 repetitions.	Synapsys force platform	Mean (SD) 95% Confidence ellipse area mm^2: NSNP 1) 262(155), 2) 708(411), 3) 330(200), and 4) 579(351); C 1) 353(346), 2) 870(760), 3) 378(217), and 4) 614(539).
**Jorgensen et al. 2011 (52)**	A cross-sectional study	Total n=194: NSNP n=85 (mNR/fNR), mean (SD) age 45(8.2); C n=109 (mNR/fNR), mean (SD) age 45(8.6).	Neck pain >30 days	Barefoot standing. Balance tasks: 1) A unilateral standing with eyes open, 2) Romberg test with feet together, arms crossed over chest and eyes open, and 3) Romberg test with feet together, arms crossed over chest, and eyes closed - test duration 30 seconds, 3 repetitions.	AMTI force platform	Mean (SD) 95% Confidence ellipse area mm^2: NSNP 1) 916(387), 2) 485(236), and 3) 884(587); C 1) 902(367), 2) 470(286), and 3) 699(386).
**Juul-Kristensen et al. 2013 (53)**	A cross-sectional study	Total n=20: WAD n=10 (m0/f10), mean (SD) age 37.70(13.64); C n=10 (m0/f10), mean (SD) age 35.90(12.45).	Chronic (≥2years)	Balance tasks: 1) Romberg stance with open, 2) Romberg stance with closed eyes, and 3) a one-legged stance - test duration 30 seconds, 3 repetitions.	AMTI force platform	Mean (SD) 95% Confidence ellipse area mm^2: WAD 1) 625.20(612.69), 2) 1,186.37(608.97), and 3) 1,276.56(387.30); C 1) 401.50(327.49), 2) 653.50(285.96), and 3) 909.87(361.23).
**Lange et al. 2014 (54)**	A randomized controlled study	Total n=55: NSNP n=30 (m30/f0), median (interquartile range) age 33.5(29-36); C n=25 (m25/f0) median (interquartile range) age 33(30-34).	Neck pain within the previous 3 months	Standing without shoes. Balance tasks: 1) Romberg position feet together, arms crossed in front of the chest and eyes open, 2) Romberg position with eyes closed, and 3) unilateral stance with one foot elevated and eyes open - test duration 30 seconds, 3 repetitions.	AMTI static force platform	Mean (SD) 95% Confidence ellipse area mm^2: NSNP 1) 485(276), 2) 697(297), and 3) 1146(447); C 1) 425(223), 2) 687(316), and 3) 1062(373).
**Michaelson et al. 2003 (51)**	A single-blind comparative group study	Total n=34: NSNP n=9 (m0/f9), mean (SD) age 40(9); WAD n=9 (m3/f6), mean (SD) age 44(10); C n=16 (m3/f13), mean (SD) age 41(9).	Chronic (≥6 months)	Barefoot standing. Balance tasks: 1) Romberg position feet together, with eyes open, 2) Romberg position feet together, with eyes closed, 3) Tandem standing heel-to-toe with eyes open, 4) Unilateral standing on the right leg, and 5) Unilateral standing on the left leg - test duration 30 seconds.	Kistler static force platform	Mean (SD) 95% Confidence ellipse area cm^2: NSNP 1) 10.5(7.3), 2) 16.6(11.7), 3) 13.1(7.2), 4) 14.8(11.), and 5) 10.6(5.6); WAD 1) 9.6(5.7), 2) 26.9(14.7), 3) 15.8(2.1), 4) 21.8(16.8), and 5) 23.1(3.3); C 1) 6.6(4.7), 2) 10.9(6.5), 3) 11.6(8.5), 4) 13.6(7.9), and 5) 10.2(3.9)
**De Pauw et al. 2018 (38)**	A case-control study	Total n=103: WAD n=35 (m0/f35), mean (SD) age 47(1.11); NSNP n=38 (m0/f38), mean (SD) age 38(1.41); C n=30 (m0/f30), mean (SD) age 30.45 (1.15)	Chronic (>3 months)	Standing, feet placed at hip width and, eyes closed - test duration 90 seconds, 3 repetitions.	AMTI ACG portable force plate	Mean (SD) 95% Confidence ellipse area cm^2: NSNP 2.72(1.66); WAD 4.11(2.88); C 1.76(0.61)

Appendix 3: Meta-analyses as forest plots for joint position error, balance and oculomotor function test

Study	HC Mean	SD	n	NSNP Mean	SD	n	Hedges' g (CI95%)	Weight
Grip et al. 2007 (29)	3.1	1.3	24	3.7	1.6	21	0.41 (-0.18, 1.01)	8.54 %
Cheever et al. 2017 (37)	3.43	1.45	18	3.38	1.53	22	-0.03 (-0.66, 0.60)	8.45 %
Elsig et al. 2014 (35)	2.78	0.87	30	3.27	1.72	30	0.35 (-0.15, 0.87)	8.77 %
Uthaikhup et al. 2012 (34)	4.2	2.2	20	5.5	3.1	20	0.47 (-0.15, 1.12)	8.44 %
Goncalves et al. 2019 (40)	3.79	1.71	33	5.12	2.67	33	0.59 (0.10, 1.09)	8.81 %
Portelli et al. 2018 (39)	3.95	1.34	22	4.27	1.49	22	0.11 (-0.37, 0.83)	8.54 %
Dugailly et al. 2015 (36)	3	1.2	36	5.3	2.5	35	1.17 (0.67, 1.69)	8.78 %
Revel et al. 1991 (24)	3.5	0.76	30	6.1	2.23	30	1.54 (0.98, 2.14)	8.58 %
Alalawi et al. 2022 (44)	3.8	2.1	15	5.5	5.9	8	0.43 (-0.44, 1.33)	7.70 %
Ghamkhar et al. 2020 (41)	9.82	3.68	30	10.99	5.53	30	0.25 (-0.26, 0.76)	8.77 %
Cid et al. 2022 (46)	2.88	0.6	14	3.03	0.9	13	0.19 (-0.57, 0.97)	8.06 %
Moustafa et al. 2022 (47)	0.35	0.2	30	2.37	0.44	30	5.83 (4.73, 7.09)	6.56 %
**Total**			**302**			**294**	**0.86 (-0.06, 1.78)**	**100 %**

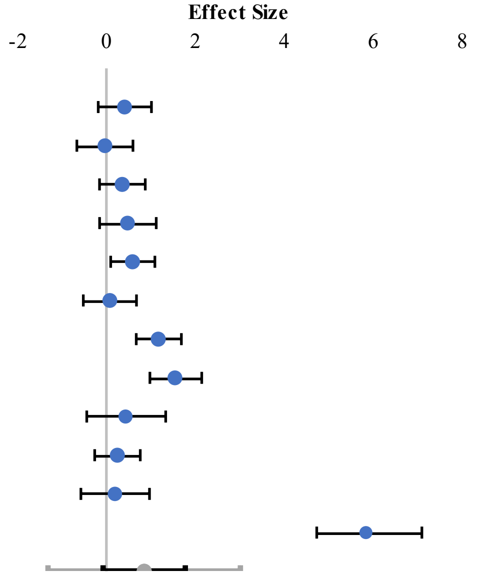

Heterogeneity: I2 = 89.43%; Test for overall effect: Z = 2.06 (p = 0.04)

Forest plot demonstrating the meta-analysis of head reposition error to neutral after cervical right rotation between people with non-specific neck pain and healthy controls: The mean results are presented in degrees, those that exceed the 4.5 cutoff are in red; CI = Confident interval; HC = Healthy controls; n = Number of subjects; SD = Standard deviation; NSNP = Non-specific neck pain.

Study	HC Mean	SD	n	NSNP Mean	SD	n	Hedges' g (CI95%)	Weight
Grip et al. 2007 (29)	3.5	1.3	24	3.6	3	21	0.04 (-0.55, 0.64)	8.58 %
Cheever et al. 2017 (37)	4.08	1.71	18	5.47	3.77	22	0.45 (-0.18, 1.10)	8.44 %
Elsig et al. 2014 (35)	2.58	0.83	30	3.1	1.15	30	0.51 (0.00, 1.04)	8.79 %
Uthaikhup et al. 2012 (34)	2.8	1.8	20	5.1	4	20	0.73 (0.09, 1.39)	8.41 %
Goncalves et al. 2019 (40)	3.87	2.1	33	5.01	3.25	33	0.41 (-0.07, 0.91)	8.87 %
Portelli et al. 2018 (39)	3.62	1.57	22	4.48	1.84	22	0.49 (-0.10, 1.11)	8.54 %
Dugailly et al. 2015 (36)	3	1.4	36	5.5	2.7	35	1.15 (0.66, 1.67)	8.82 %
Revel et al. 1991 (24)	3.5	1.14	30	6.11	2.1	30	1.52 (0.96, 2.13)	8.60 %
Alalawi et al. 2022 (44)	4.2	2.8	15	5.2	5.2	8	0.26 (-0.62, 1.15)	7.69 %

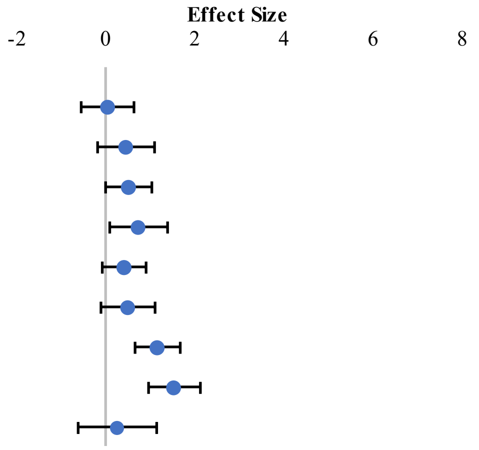

Ghamkhar et al. 2020 (41)	9.06	3.51	30	11.95	4.98	30	0.66 (0.15, 1.20)	8.78 %	
Cid et al. 2022 (46)	3.03	0.7	14	3.67	1.8	13	0.46 (-0.30, 1.26)	8.01 %	
Moustafa et al. 2022 (47)	0.35	0.2	30	2.37	0.44	30	5.83 (4.73, 7.09)	6.47 %	
**Total**			**302**			**294**	**0.95 (0.06, 1.84)**	**100 %**	

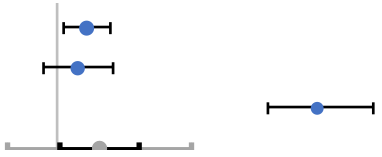

Heterogeneity: I2 = 88.54%; Test for overall effect: Z = 2.36 (p = 0.02)

Forest plot demonstrating the meta-analysis of head reposition error to neutral after cervical left rotation between people with non-specific neck pain and healthy controls: The mean results are presented in degrees, those that exceed the 4.5 cutoff are in red; CI = Confident interval; HC = Healthy controls; n = Number of subjects; SD = Standard deviation; NSNP = Non-specific neck pain.

	HC			NSNP				
**Study**	**Mean**	**SD**	**n**	**Mean**	**SD**	**n**	**Hedges' g (CI95%)**	**Weight**
Lopez-de-Uralde-Villanueva et al. 2020 (42	3.89	1.49	48	4.54	2.59	68	0.29 (-0.08, 0.67)	15.31 %
Kristjansson et al. 2003 (26)	2.48	1.12	21	3.33	1.42	20	0.65 (0.03, 1.30)	13.25 %
Woodhouse et al. 2008 (30)	2.86	1.2	57	3.17	1.1	57	0.27 (-0.10, 0.64)	15.33 %
Van den Oord et al. 2010 (33)	1.8	0.6	61	1.9	0.6	17	0.17 (-0.37, 0.71)	13.99 %
De Pauw et al. 2018 (38)	3.46	1.44	30	3.81	1.47	38	0.24 (-0.24, 0.72)	14.49 %
Revel et al. 1991 (24)	3.5	0.82	30	6.11	1.59	30	2.04 (1.43, 2.69)	13.23 %
Roren et al. 2009 (32)	3.6	0.8	41	6.3	2.4	41	1.5 (1.02, 2.00)	14.39 %
**Total**			**288**			**271**	**0.71 (0.04, 1.39)**	**100 %**

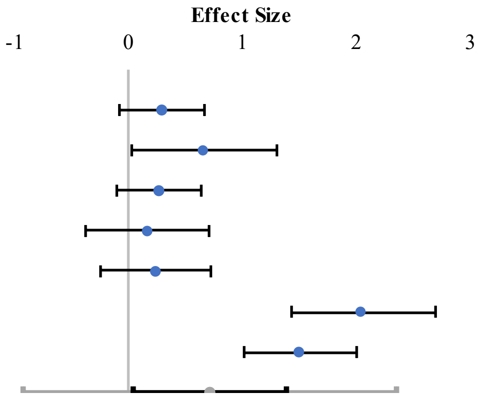

Heterogeneity: I2 = 86.49%; Test for overall effect: Z = 2.60 (p < 0.01)

Forest plot demonstrating the meta-analysis of head reposition error to neutral after cervical rotation between people with non-specific neck pain and healthy controls: The mean results are presented in degrees, those that exceed the 4.5 cutoff are in red; CI = Confident interval; HC = Healthy controls; n = Number of subjects; SD = Standard deviation; NSNP = Non-specific neck pain.

	HC			NSNP				
**Study**	**Mean**	**SD**	**n**	**Mean**	**SD**	**n**	**Hedges' g (CI95%)**	**Weight**
Lopez-de-Uralde-Villanueva et al. 2020 (42	3.97	1.5	48	4.1	2.32	68	0.06 (-0.31, 0.44)	13.57 %
Grip et al. 2007 (29)	2.9	0.9	24	2.8	1.2	21	-0.09 (-0.69, 0.50)	12.34 %
Cheever et al. 2017 (37)	2.74	1.52	18	4.53	1.71	22	1.08 (0.42, 1.78)	11.81 %
Elsig et al. 2014 (35)	2.67	0.98	30	3.43	1.75	30	0.53 (0.02, 1.06)	12.77 %
Portelli et al. 2018 (39)	2.95	1.17	22	3.91	1.44	22	0.72 (0.11, 1.35)	12.18 %
Dugailly et al. 2015 (36)	3.1	1.7	36	5.1	2.6	35	0.90 (0.42, 1.41)	12.92 %
Revel et al. 1991 (24)	3.31	1.14	30	5.48	0.23	30	2.60 (1.94, 3.34)	11.61 %

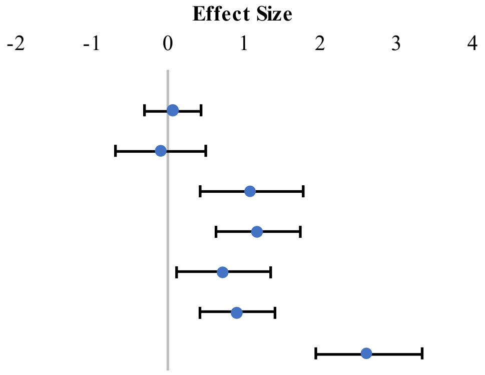

Ghamkhar et al. 2020 (41)	8.65	3.14	30	9.15	3.59	30	0.15 (-0.36, 0.66)	12.82 %
**Total**			**238**			**258**	**0.72 (0.01, 1.42)**	**100 %**

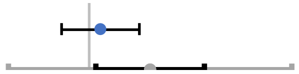

Heterogeneity: I2 = 86.95%; Test for overall effect: Z = 2.41 (p = 0.02)

Forest plot demonstrating the meta-analysis of head reposition error to neutral after cervical flexion between people with non-specific neck pain and healthy controls: The mean results are presented in degrees, those that exceed the 4.5 cutoff are in red; CI = Confident interval; HC = Healthy controls; n = Number of subjects; SD = Standard deviation; NSNP = Non-specific neck pain.

	HC			NSNP				
**Study**	**Mean**	**SD**	**n**	**Mean**	**SD**	**n**	**Hedges' g (CI95%)**	**Weight**
Lopez-de-Uralde-Villanueva et al. 2020 (42	4.24	2.37	48	5.21	3.78	68	0.29 (-0.08, 0.67)	13.51 %
Grip et al. 2007 (29)	2.7	1	24	2.9	1.3	21	0.17 (-0.42, 0.77)	10.58 %
Cheever et al. 2017 (37)	3.78	1.95	18	5.77	2.73	22	0.81 (0.17, 1.48)	9.80 %
Elsig et al. 2014 (35)	2.65	0.95	30	3.19	1.31	30	0.47 (-0.05, 0.99)	11.55 %
Portelli et al. 2018 (39)	3.35	1.46	22	3.98	1.85	22	0.37 (-0.22, 0.98)	10.46 %
Dugailly et al. 2015 (36)	3.5	1.3	36	7.3	3.4	35	1.47 (0.96, 2.01)	11.36 %
Revel et al. 1991 (24)	3.43	0.82	30	5.47	2.29	30	1.17 (0.63, 1.74)	11.06 %
Uthaikhup et al. 2012 (34)	3.6	2.4	20	5.2	3.4	20	0.53 (-0.10, 1.18)	10.02 %
Ghamkhar et al. 2020 (41)	10.82	3.27	30	11.24	4.5	30	0.11 (-0.40, 0.62)	11.64 %
**Total**			**258**			**278**	**0.59 (0.23, 0.95)**	**100 %**

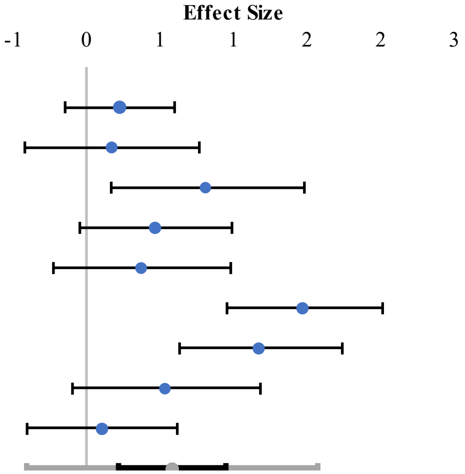

Heterogeneity: I2 = 67.55%; Test for overall effect: Z = 3.80 (p < 0.01)

Forest plot demonstrating the meta-analysis of head reposition error to neutral after cervical extension between people with non-specific neck pain and healthy controls: The mean results are presented in degrees, those that exceed the 4.5 cutoff are in red; CI = Confident interval; HC = Healthy controls; n = Number of subjects; SD = Standard deviation; NSNP = Non-specific neck pain.

	HC			NSNP				
**Study**	**Mean**	**SD**	**n**	**Mean**	**SD**	**n**	**Hedges' g (CI95%)**	**Weight**
Van den Oord et al. 2010 (33)	3.1	1.2	61	2.8	1	17	-0.26 (-0.80, 0.28)	33.27 %
De Pauw et al. 2018 (38)	3.04	0.99	30	3.5	1.2	38	0.41 (-0.07, 0.90)	33.93 %
Revel et al. 1991 (24)	3.37	0.73	30	5.47	1.75	30	1.55 (0.98, 2.15)	32.80 %
**Total**			**121**			**85**	**0.56 (-1.69, 2.81)**	**100 %**

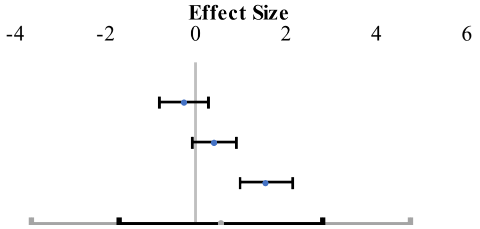

Heterogeneity: I2 = 90.35%; Test for overall effect: Z = 1.07 (p = 0.28)

Forest plot demonstrating the meta-analysis of head reposition error to neutral after cervical flexion-extension between people with non-specific neck pain and healthy controls: The mean results are presented in degrees, those that exceed the 4.5 cutoff are in red; CI = Confident interval; HC = Healthy controls; n = Number of subjects; SD = Standard deviation; NSNP = Non-specific neck pain.

	HC			TNPDM				
**Study**	**Mean**	**SD**	**n**	**Mean**	**SD**	**n**	**Hedges' g (CI95%)**	**Weight**
Grip et al. 2007 (29)	3.5	1.3	24	4	2.1	22	0.28 (-0.30, 0.88)	12.69 %
Sterling et al. 2004 (28)	2.3	1.34	20	2.4	1.2	36	0.08 (-0.47, 0.63)	12.82 %
Treleaven et al. 2003 (12)	2	1.33	44	3.9	2.62	76	0.85 (0.46, 1.24)	13.34 %
Hill et al. 2009 (31)	2.47	2.83	50	4.01	2.12	50	0.61 (0.21, 1.02)	13.30 %
Heikkilä et al. 1998 (25)	2.69	1.78	34	3.99	3	14	0.58 (-0.05, 1.23)	12.49 %
Sterling et al. 2003 (27)	2.6	1.34	20	2.7	0.94	22	0.09 (-0.53, 0.70)	12.60 %
Alalawi et al. 2022 (45)	3.1	2.6	42	3.8	2.4	18	0.27 (-0.28, 0.83)	12.79 %
Moustafa et al. 2022 (47)	0.35	0.2	30	3.03	0.61	30	5.83 (4.72, 7.08)	9.96 %
**Total**			**264**			**268**	**0.94 (-0.53, 2.41)**	**100 %**

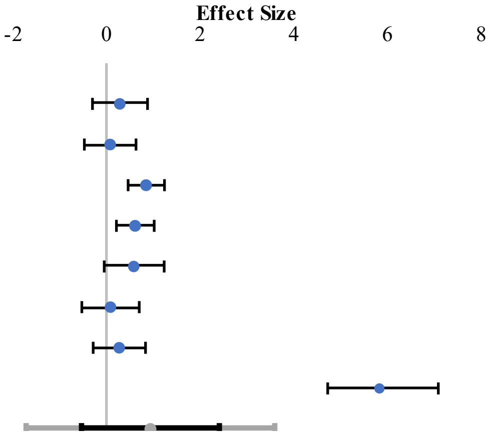

Heterogeneity: I2 = 92.15%; Test for overall effect: Z = 1.51 (p = 0.13)

Forest plot demonstrating the meta-analysis of head reposition error to neutral after cervical left rotation between people with traumatic neck pain with dizziness and mild pain, and healthy controls: The mean results are presented in degrees; CI = Confident interval; HC = Healthy controls; n = Number of subjects; SD = Standard deviation; TNPDM = Traumatic neck pain with dizziness and mild pain.

	HC			TNPNDM				
**Study**	**Mean**	**SD**	**n**	**Mean**	**SD**	**n**	**Hedges' g (CI95%)**	**Weight**
Grip et al. 2007 (29)	3.5	1.3	24	4	2.1	22	0.28 (-0.30, 0.88)	12.72 %
Sterling et al. 2004 (28)	2.3	1.34	20	2.4	1.2	36	0.08 (-0.47, 0.63)	12.84 %
Treleaven et al. 2003 (12)	2	1.33	44	2.8	2.04	26	0.49 (0.00, 0.99)	13.03 %
Hill et al. 2009 (31)	2.47	2.83	50	3.07	2.12	50	0.24 (-0.16, 0.64)	13.32 %
Heikkilä et al. 1998 (25)	2.69	1.78	34	3.99	3	14	0.58 (-0.05, 1.23)	12.53 %
Sterling et al. 2003 (27)	2.6	1.34	20	2.7	0.94	22	0.09 (-0.53, 0.70)	12.64 %
Alalawi et al. 2022 (45)	3.1	2.6	42	3.8	2.4	18	0.27 (-0.28, 0.83)	12.81 %
Moustafa et al. 2022 (47)	0.35	0.2	30	3.03	0.61	30	5.83 (4.72, 7.08)	10.11 %
**Total**			**264**			**218**	**0.85 (-0.65, 2.35)**	**100 %**

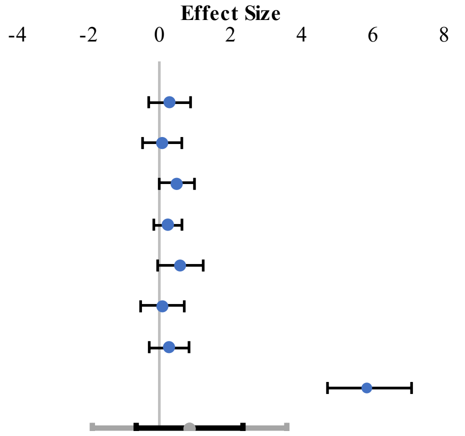

Heterogeneity: I2 = 92.08%; Test for overall effect: Z = 1.34 (p = 0.18)

Forest plot demonstrating the meta-analysis of head reposition error to neutral after cervical left rotation between people with traumatic neck pain with non-dizziness and mild pain, and healthy controls: The mean results are presented in degrees; CI = Confident interval; HC = Healthy controls; n = Number of subjects; SD = Standard deviation; TNPNDM = Traumatic neck pain with non-dizziness and mild pain.

	HC			TNPDS				
**Study**	**Mean**	**SD**	**n**	**Mean**	**SD**	**n**	**Hedges' g (CI95%)**	**Weight**
Grip et al. 2007 (29)	3.5	1.3	24	4	2.1	22	0.28 (-0.30, 0.88)	12.81 %
Sterling et al. 2004 (28)	2.3	1.34	20	3.3	1.73	12	0.65 (-0.08, 1.41)	12.18 %
Treleaven et al. 2003 (12)	2	1.33	44	3.9	2.62	76	0.85 (0.46, 1.24)	13.48 %
Hill et al. 2009 (31)	2.47	2.83	50	4.01	2.12	50	0.61 (0.21, 1.02)	13.44 %
Heikkilä et al. 1998 (25)	2.69	1.78	34	3.99	3	14	0.58 (-0.05, 1.23)	12.59 %
Sterling et al. 2003 (27)	2.6	1.34	20	3.2	1.31	19	0.44 (-0.19, 1.10)	12.59 %
Alalawi et al. 2022 (45)	3.1	2.6	42	3.8	2.4	18	0.27 (-0.28, 0.83)	12.91 %
Moustafa et al. 2022 (47)	0.35	0.2	30	3.03	0.61	30	5.83 (4.72, 7.08)	10.00 %
**Total**			**264**			**241**	**1.06 (-0.37, 2.49)**	**100 %**

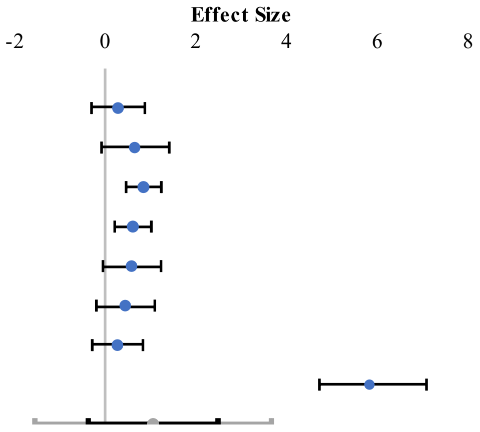

Heterogeneity: I2 = 91.41%; Test for overall effect: Z = 1.75 (p = 0.08)

Forest plot demonstrating the meta-analysis of head reposition error to neutral after cervical left rotation between people with traumatic neck pain with dizziness and severe pain, and healthy controls: The mean results are presented in degrees; CI = Confident interval; HC = Healthy controls; n = Number of subjects; SD = Standard deviation; TNPDS = Traumatic neck pain with dizziness and severe pain.

	HC			TNPNDS				
**Study**	**Mean**	**SD**	**n**	**Mean**	**SD**	**n**	**Hedges' g (CI95%)**	**Weight**
Grip et al. 2007 (29)	3.5	1.3	24	4	2.1	22	0.28 (-0.30, 0.88)	12.82 %
Sterling et al. 2004 (28)	2.3	1.34	20	3.3	1.73	12	0.65 (-0.08, 1.41)	12.25 %
Treleaven et al. 2003 (12)	2	1.33	44	2.8	2.04	26	0.49 (0.00, 0.99)	13.12 %
Hill et al. 2009 (31)	2.47	2.83	50	3.07	2.12	50	0.24 (-0.16, 0.64)	13.40 %
Heikkilä et al. 1998 (25)	2.69	1.78	34	3.99	3	14	0.58 (-0.05, 1.23)	12.63 %
Sterling et al. 2003 (27)	2.6	1.34	20	3.2	1.31	19	0.44 (-0.19, 1.10)	12.63 %
Alalawi et al. 2022 (45)	3.1	2.6	42	3.8	2.4	18	0.27 (-0.28, 0.83)	12.91 %
Moustafa et al. 2022 (47)	0.35	0.2	30	3.03	0.61	30	5.83 (4.72, 7.08)	10.24 %
**Total**			**264**			**191**	**0.97 (-0.50, 2.44)**	**100 %**

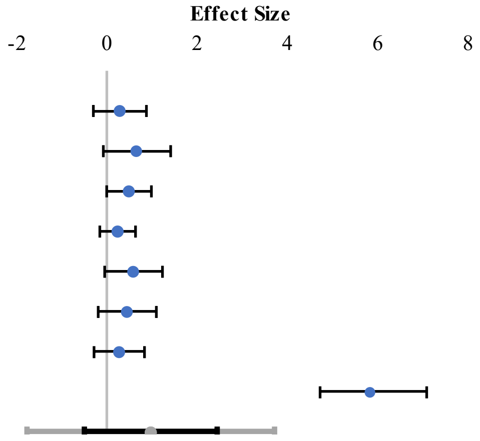

Heterogeneity: I2 = 91.72%; Test for overall effect: Z = 1.56 (p = 0.19)

Forest plot demonstrating the meta-analysis of head reposition error to neutral after cervical left rotation between people with traumatic neck pain with non-dizziness and severe pain, and healthy controls: The mean results are presented in degrees; CI = Confident interval; HC = Healthy controls; n = Number of subjects; SD = Standard deviation; TNPNDS = Traumatic neck pain with non-dizziness and severe pain.

	HC			TNPDM				
**Study**	**Mean**	**SD**	**n**	**Mean**	**SD**	**n**	**Hedges' g (CI95%)**	**Weight**
Grip et al. 2007 (29)	3.1	1.3	24	3.7	1.9	22	0.37 (-0.22, 0.96)	12.70 %
Sterling et al. 2004 (28)	2.3	2.24	20	2.6	1.8	36	0.15 (-0.40, 0.70)	12.82 %
Treleaven et al. 2003 (12)	2.5	1.33	44	4.5	2.62	76	0.89 (0.51, 1.29)	13.32 %
Hill et al. 2009 (31)	3.16	2.83	50	4.55	2.83	50	0.49 (0.09, 0.89)	13.30 %
Heikkilä et al. 1998 (25)	2.78	2	34	4.32	2.86	14	0.67 (0.03, 1.32)	12.49 %
Sterling et al. 2003 (27)	2.7	1.34	20	2.7	1.41	22	0.00 (-0.61, 0.61)	12.62 %
Alalawi et al. 2022 (45)	3.2	2.1	42	3.4	2.1	18	0.09 (-0.46, 0.65)	12.80 %
Moustafa et al. 2022 (47)	0.35	0.2	30	3.10	0.611	30	5.97 (4.84, 7.25)	9.94 %
**Total**			**264**			**268**	**0.94 (-0.58, 2.45)**	**100 %**

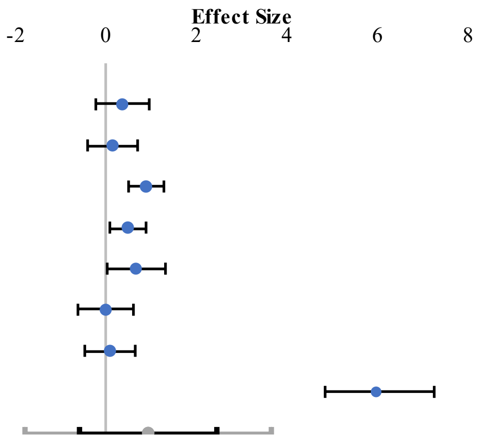

Heterogeneity: I2 = 92.71%; Test for overall effect: Z = 1.46 (p = 0.14)

Forest plot demonstrating the meta-analysis of head reposition error to neutral after cervical right rotation between people with traumatic neck pain with dizziness and mild pain, and healthy controls: The mean results are presented in degrees, those that exceed the 4.5 cutoff are in red; CI = Confident interval; HC = Healthy controls; n = Number of subjects; SD = Standard deviation; TNPDM = Traumatic neck pain with dizziness and mild pain.

	HC			TNPNDM				
**Study**	**Mean**	**SD**	**n**	**Mean**	**SD**	**n**	**Hedges' g (CI95%)**	**Weight**
Grip et al. 2007 (29)	3.1	1.3	24	3.7	1.9	22	0.37 (-0.22, 0.96)	12.72 %
Sterling et al. 2004 (28)	2.3	2.24	20	2.6	1.8	36	0.15 (-0.40, 0.70)	12.83 %
Treleaven et al. 2003 (12)	2.5	1.33	44	2.9	2.04	26	0.24 (-0.24, 0.74)	13.02 %
Hill et al. 2009 (31)	3.16	2.83	50	2.93	2.83	50	-0.08 (-0.48, 0.31)	13.28 %
Heikkilä et al. 1998 (25)	2.78	2	34	4.32	2.86	14	0.67 (0.03, 1.32)	12.53 %
Sterling et al. 2003 (27)	2.7	1.34	20	2.7	1.41	22	0.00 (-0.61, 0.61)	12.65 %
Alalawi et al. 2022 (45)	3.2	2.1	42	3.4	2.1	18	0.09 (-0.46, 0.65)	12.81 %
Moustafa et al. 2022 (47)	0.35	0.2	30	3.1	0.61	30	5.97 (4.84. 7.25)	10.16 %
**Total**			**264**			**218**	**0.79 (-0.78, 2.36)**	**100 %**

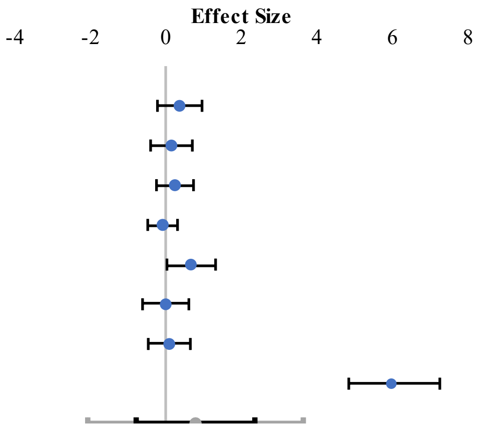

Heterogeneity: I2 = 92.69%; Test for overall effect: Z = 1.19 (p = 0.24)

Forest plot demonstrating the meta-analysis of head reposition error to neutral after cervical right rotation between people with traumatic neck pain with non-dizziness and mild pain, and healthy controls: The mean results are presented in degrees; CI = Confident interval; HC = Healthy controls; n = Number of subjects; SD = Standard deviation; TNPNDM = Traumatic neck pain with non-dizziness and mild pain.

	HC			TNPDS				
Study	Mean	SD	n	Mean	SD	n	Hedges' g (CI95%)	Weight
Grip et al. 2007 (29)	3.1	1.3	24	3.7	1.9	22	0.37 (-0.22, 0.96)	12.84 %
Sterling et al. 2004 (28)	2.3	2.24	20	4.5	2.42	12	0.93 (0.19, 1.72)	12.21 %
Treleaven et al. 2003 (12)	2.5	1.33	44	4.5	2.62	76	0.89 (0.51, 1.29)	13.44 %
Hill et al. 2009 (31)	3.16	2.83	50	4.55	2.83	50	0.49 (0.09, 0.89)	13.41 %
Heikkilä et al. 1998 (25)	2.78	2	34	4.32	2.86	14	0.67 (0.03, 1.32)	12.64 %
Sterling et al. 2003 (27)	2.7	1.34	20	4.8	1.31	19	1.55 (0.86, 2.31)	12.33 %
Alalawi et al. 2022 (45)	3.2	2.1	42	3.4	2.1	18	0.09 (-0.46, 0.65)	12.94 %
Moustafa et al. 2022 (47)	0.35	0.2	30	3.1	0.61	30	5.97 (4.84, 7.25)	10.18 %
**Total**			**264**			**241**	**1.24 (-0.23, 2.71)**	**100 %**

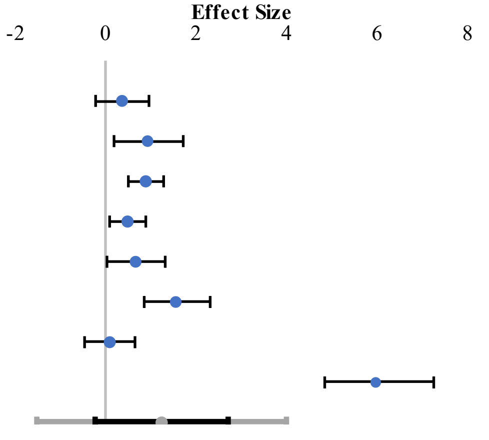

Heterogeneity: I2 = 92.20%; Test for overall effect: Z = 2.00 (p = 0.05)

Forest plot demonstrating the meta-analysis of head reposition error to neutral after cervical right rotation between people with traumatic neck pain with dizziness and severe pain, and healthy controls: The mean results are presented in degrees, those that exceed the 4.5 cutoff are in red; CI = Confident interval; HC = Healthy controls; n = Number of subjects; SD = Standard deviation; TNPDS = Traumatic neck pain with dizziness and severe pain.

	HC			TNPNDS				
Study	Mean	SD	n	Mean	SD	n	Hedges' g (CI95%)	Weight
Grip et al. 2007 (29)	3.1	1.3	24	3.7	1.9	22	0.37 (-0.22, 0.96)	12.81 %
Sterling et al. 2004 (28)	2.3	2.24	20	4.5	2.42	12	0.93 (0.19, 1.72)	12.31 %
Treleaven et al. 2003 (12)	2.5	1.33	44	2.9	2.04	26	0.24 (-0.24, 0.74)	13.06 %
Hill et al. 2009 (31)	3.16	2.83	50	2.93	2.83	50	-0.08 (-0.48, 0.31)	13.27 %
Heikkilä et al. 1998 (25)	2.78	2	34	4.32	2.86	14	0.67 (0.03, 1.32)	12.65 %
Sterling et al. 2003 (27)	2.7	1.34	20	4.8	1.31	19	1.55 (0.86, 2.31)	12.40 %
Alalawi et al. 2022 (45)	3.2	2.1	42	3.4	2.1	18	0.09 (-0.46, 0.65)	12.89 %
Moustafa et al. 2022 (47)	0.35	0.2	30	3.1	0.61	30	5.97 (4.84, 7.25)	10.60 %
**Total**			**264**			**191**	**1.10 (-0.46, 2.66)**	**100 %**

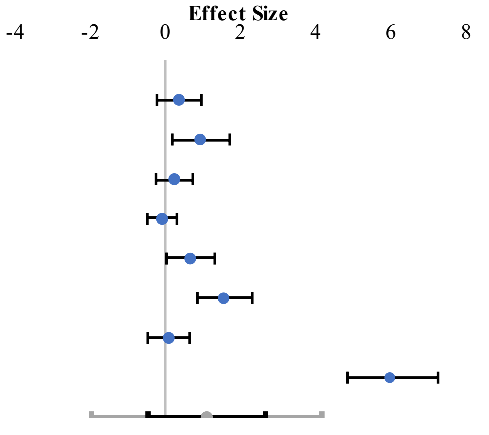

Heterogeneity: I2 = 93.32%; Test for overall effect: Z = 1.67 (p = 0.09)

Forest plot demonstrating the meta-analysis of head reposition error to neutral after cervical right rotation between people with traumatic neck pain with non-dizziness and severe pain, and healthy controls: The mean results are presented in degrees, those that exceed the 4.5 cutoff are in red; CI = Confident interval; HC = Healthy controls; n = Number of subjects; SD = Standard deviation; TNPNDS = Traumatic neck pain with non-dizziness and severe pain.

	HC			TNP				
**Study**	**Mean**	**SD**	**n**	**Mean**	**SD**	**n**	**Hedges' g (CI95%)**	**Weight**
Kristjansson et al 2003 (26)	2.48	1.12	21	4.14	1.58	22	1.19 (0.55, 1.86)	26.16 %
Woodhouse et al 2008 (30)	2.86	1.2	57	3.35	1.6	59	0.34 (-0.02, 0.71)	40.41 %
De Pauw et al 2018 (38)	3.46	1.44	30	4.3	2.16	35	0.45 (-0.05, 0.95)	33.43 %
**Total**			**108**			**116**	**0.60 (-0.47, 1.67)**	**100 %**

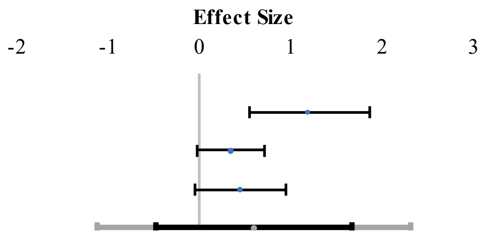

Heterogeneity: I2 = 61.28%; Test for overall effect: Z = 2.40 (p = 0.02)

Forest plot demonstrating the meta-analysis of head reposition error to neutral after cervical rotation between people with traumatic neck pain and healthy controls: The mean results are presented in degrees; CI = Confident interval; HC = Healthy controls; n = Number of subjects; SD = Standard deviation; TNP = Traumatic neck pain.

	HC			TNPDM				
**Study**	**Mean**	**SD**	**n**	**Mean**	**SD**	**n**	**Hedges' g (CI95%)**	**Weight**
Sterling et al 2004 (28)	2.9	2.68	20	3.6	2.4	36	0.28 (-0.27, 0.83)	13.48 %
Treleaven et al 2003 (12)	2.4	1.99	44	3.5	2.62	76	0.45 (0.08, 0.83)	28.35 %
Hill et al 2008 (31)	3.01	2.12	50	3.61	2.12	50	0.28 (-0.11, 0.68)	25.86 %
Heikkilä et al 1998 (25)	2.84	1.84	34	5.21	3.46	14	0.97 (0.32, 1.64)	9.59 %
Sterling et al 2003 (27)	2.8	1.34	20	3.4	1.41	22	0.43 (-0.18, 1.06)	10.94 %
Grip et al 2007 (29)	2.7	1	24	3.5	1.8	22	0.55 (-0.04, 1.15)	11.77 %
**Total**			**192**			**220**	**0.44 (0.22, 0.67)**	**100 %**

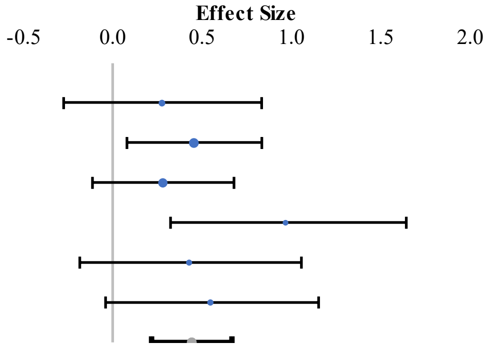

Heterogeneity: I2 = 0%; Test for overall effect: Z = 5.06 (p < 0.01)

Forest plot demonstrating the meta-analysis of head reposition error to neutral after cervical between people with traumatic neck pain with dizziness and mild pain, and healthy controls: The mean results are presented in degrees, those that exceed the 4.5 cutoff are in red; CI = Confident interval; HC = Healthy controls; n = Number of subjects; SD = Standard deviation; TNPDM = Traumatic neck pain with dizziness and mild pain.

	HC			TNPNDM				
**Study**	**Mean**	**SD**	**n**	**Mean**	**SD**	**n**	**Hedges' g (CI95%)**	**Weight**
Sterling et al 2004 (28)	2.9	2.6833	20	3.6	2.4	36	0.28 (-0.27, 0.83)	16.38 %
Treleaven et al 2003 (12)	2.4	1.99	44	3.5	2.0396	26	0.54 (0.05, 1.04)	18.26 %
Hill et al 2008 (31)	3.01	2.1213	50	2.84	2.1213	50	-0.08 (-0.47, 0.31)	22.27 %
Heikkilä et al 1998 (25)	2.84	1.84	34	5.21	3.46	14	0.97 (0.32, 1.64)	13.40 %
Sterling et al 2003 (27)	2.8	1.3416	20	3.4	1.4071	22	0.43 (-0.18, 1.06)	14.53 %
Grip et al 2007 (29)	2.7	1	24	3.5	1.8	22	0.55 (-0.04, 1.15)	15.17 %

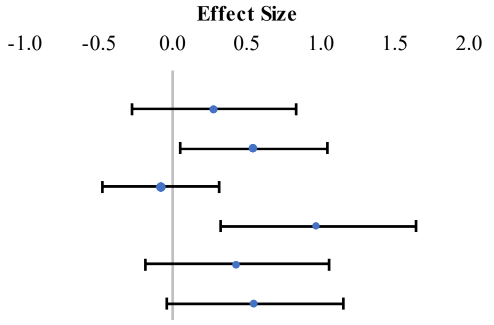

**Total**			**192**			**170**	**0.40 (0.03, 0.77)**	**100 %**

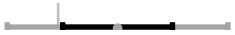

Heterogeneity: I2 = 46.80%; Test for overall effect: Z = 2.78 (p < 0.01)

Forest plot demonstrating the meta-analysis of head reposition error to neutral after cervical extension between people with traumatic neck pain with non-dizziness and mild pain, and healthy controls: The mean results are presented in degrees, those that exceed the 4.5 cutoff are in red; CI = Confident interval; HC = Healthy controls; n = Number of subjects; SD = Standard deviation; TNPNDM = Traumatic neck pain with non-dizziness and mild pain.

	HC			TNPDS				
**Study**	**Mean**	**SD**	**n**	**Mean**	**SD**	**n**	**Hedges' g (CI95%)**	**Weight**
Sterling et al 2004 (28)	2.9	2.68	20	5.4	3.12	12	0.85 (0.12, 1.64)	9.15 %
Treleaven et al 2003 (12)	2.4	1.99	44	3.5	2.62	76	0.45 (0.08, 0.83)	28.12 %
Hill et al 2008 (31)	3.01	2.12	50	3.61	2.12	50	0.28 (-0.11, 0.68)	26.25 %
Heikkilä et al 1998 (25)	2.84	1.84	34	5.21	3.46	14	0.97 (0.32, 1.64)	11.49 %
Sterling et al 2003 (27)	2.8	1.34	20	4.1	1.31	19	0.96 (0.31, 1.65)	11.21 %
Grip et al 2007 (29)	2.7	1	24	3.5	1.8	22	0.55 (-0.04, 1.15)	13.77 %
**Total**			**192**			**193**	**0.57 (0.27, 0.87)**	**100 %**

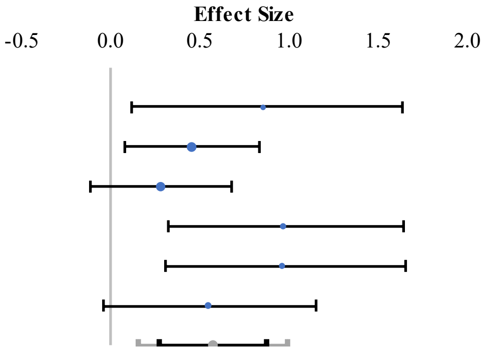

Heterogeneity: I2 = 15.24%; Test for overall effect: Z = 4.90 (p < 0.01)

Forest plot demonstrating the meta-analysis of head reposition error to neutral after cervical extension between people with traumatic neck pain with dizziness and severe pain, and healthy controls: The mean results are presented in degrees, those that exceed the 4.5 cutoff are in red; CI = Confident interval; HC = Healthy controls; n = Number of subjects; SD = Standard deviation; TNPDS = Traumatic neck pain with dizziness and severe pain.

	HC			TNPNDS				
**Study**	**Mean**	**SD**	**n**	**Mean**	**SD**	**n**	**Hedges' g (CI95%)**	**Weight**
Sterling et al 2004 (28)	2.9	2.68	20	5.4	3.12	12	0.85 (0.12, 1.64)	13.46 %
Treleaven et al 2003 (12)	2.4	1.99	44	3.5	2.04	26	0.54 (0.05, 1.04)	18.70 %
Hill et al 2008 (31)	3.01	2.12	50	2.84	2.12	50	-0.08 (-0.47, 0.31)	21.13 %
Heikkilä et al 1998 (25)	2.84	1.84	34	5.21	3.46	14	0.97 (0.32, 1.64)	15.18 %
Sterling et al 2003 (27)	2.8	1.34	20	4.1	1.31	19	0.96 (0.31, 1.65)	14.99 %
Grip et al 2007 (29)	2.7	1	24	3.5	1.8	22	0.55 (-0.04, 1.15)	16.54 %
**Total**			**192**			**143**	**0.58 (0.14, 1.02)**	**100 %**

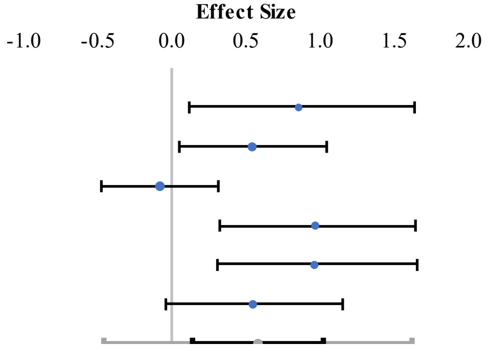

Heterogeneity: I2 = 62.60%; Test for overall effect: Z = 3.39 (p < 0.01)

Forest plot demonstrating the meta-analysis of head reposition error to neutral after cervical extension between people with traumatic neck pain with non-dizziness and severe pain, and healthy controls: The mean results are presented in degrees, those that exceed the 4.5 cutoff are in red; CI = Confident interval; HC = Healthy controls; n = Number of subjects; SD = Standard deviation; TNPNDS = Traumatic neck pain with non-dizziness and severe pain.

	NSNP			TNP				
Study	Mean	SD	n	Mean	SD	n	Hedges' g (CI95%)	Weight
Kristjansson et al 2003 (26)	3.33	1.42	20	4.14	1.58	22	0.53 (-0.09, 1.16)	18.03 %
Woodhouse et al 2008 (30)	3.17	1.1	57	3.35	1.6	59	0.13 (-0.24, 0.50)	50.31 %
De Pauw et al 2018 (38)	3.81	1.47	38	4.3	2.16	35	0.26 (-0.20, 0.73)	31.66 %
**Total**			**115**			**116**	**0.24 (-0.20, 0.69)**	**100 %**

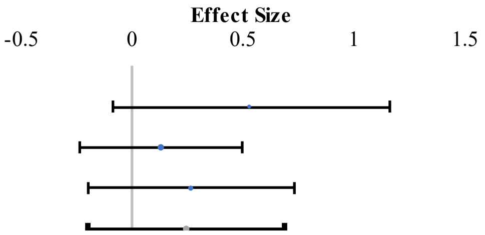

Heterogeneity: I2 = 0.00%; Test for overall effect: Z = 2.37 (p = 0.02)

Forest plot demonstrating the meta-analysis of head reposition error to neutral after cervical rotation between people with non-specific and traumatic neck pain: The mean results are presented in degrees; CI = Confident interval; n = Number of subjects; NSNP = Non-specific neck pain; SD = Standard deviation; TNP = Traumatic neck pain.

	NPD			HC				
Study	Mean	SD	n	Mean	SD	n	Hedges' g (CI95%)	Weight
Treleaven et al. 2003 (12)	4.5	2.62	76	2.5	1.33	44	-0.89 (-1.29, -0.51)	33.37 %
Hill et al. 2009 (31)	4.55	2.83	50	3.16	2.83	50	-0.49 (-0.89, -0.09)	33.33 %
Micarelli et al. 2020 (43)	5.32	1.26	93	2.43	0.66	98	-2.88 (-3.30, -2.49)	33.29 %
**Total**			**219**			**192**	**-1.42 (-4.61, 1.77)**	**100 %**

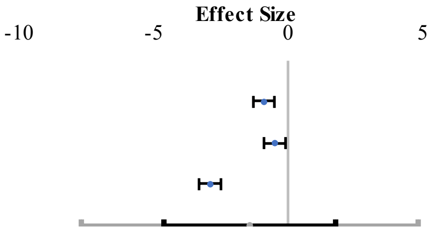

Heterogeneity: I2 = 97.47%; Test for overall effect: Z = -1.92 (p = 0.06)

Forest plot demonstrating the meta-analysis of head reposition error to neutral after cervical right rotation between people with neck pain with dizziness and healthy controls: The mean results are presented in degrees, those that exceed the 4.5 cutoff are in red; CI = Confident interval; HC = Healthy controls; n = Number of subjects; NPD = Neck pain with dizziness; SD = Standard deviation.

	NPD			HC				
Study	Mean	SD	n	Mean	SD	n	Hedges' g (CI95%)	Weight
Treleaven et al. 2003 (12)	3.9	2.6153	76	2	1.3266	44	-0.85 (-1.24, -0.46)	33.39 %
Hill et al. 2009 (31)	4.01	2.1213	50	2.47	2.8284	50	-0.61 (-1.02, -0.21)	33.34 %
Micarelli et al. 2020 (43)	5.05	1.04	93	2.48	0.61	98	-3.02 (-3.45, -2.62)	33.27 %
**Total**			**219**			**192**	**-1.49 (-4.79, 1.81)**	**100 %**

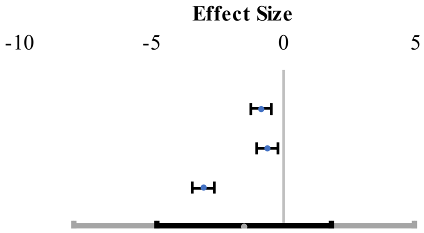

Heterogeneity: I2 = 97.56%; Test for overall effect: Z = -1.94 (p = 0.05)

Forest plot demonstrating the meta-analysis of head reposition error to neutral after cervical left rotation between people with neck pain with dizziness and healthy controls: The mean results are presented in degrees, those that exceed the 4.5 cutoff are in red; CI = Confident interval; HC = Healthy controls; n = Number of subjects; NPD = Neck pain with dizziness; SD = Standard deviation.

Study	NPD Mean	SD	n	HC Mean	SD	n	Hedges' g (CI95%)	Weight
Treleaven et al. 2003 (12)	3.5	2.6153	76	2.4	1.99	44	-0.45 (-0.83, -0.08)	33.36 %
Hill et al. 2009 (31)	3.61	2.1213	50	3.01	2.1213	50	-0.28 (-0.68, 0.11)	33.28 %
Micarelli et al. 2020 (43)	4.97	1.23	93	2.63	0.62	98	-2.41 (-2.80, -2.05)	33.36 %
**Total**			**219**			**192**	**-1.05 (-3.99, 1.89)**	**100 %**

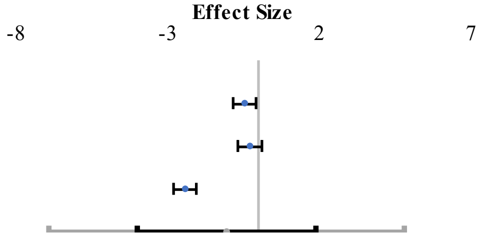

Heterogeneity: I2 = 97.37%; Test for overall effect: Z = -1.54 (p = 0.13)

Forest plot demonstrating the meta-analysis of head reposition error to neutral after cervical extension between people with neck pain with dizziness and healthy controls: The mean results are presented in degrees, those that exceed the 4.5 cutoff are in red; CI = Confident interval; HC = Healthy controls; n = Number of subjects; NPD = Neck pain with dizziness; SD = Standard deviation.

Study	HC Mean	SD	n	NSNP Mean	SD	n	Hedges' g (CI95%)	Weight
De Pauw et al. 2018 (38)	176	61	30	272	166	38	0.73 (0.24, 1.23)	27.81 %
Jorgensen et al. 2011 (52)	699	386	109	884	699	85	0.34 (0.05, 0.63)	46.67 %
Lange et al. 2014 (54)	687	316	25	697	297	30	0.03 (-0.50, 0.57)	25.52 %
**Total**			**164**			**153**	**0.37 (-0.41, 1.14)**	**100 %**

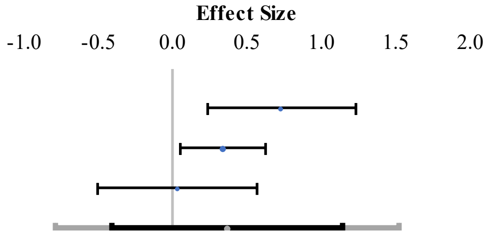

Heterogeneity: I2 = 45.74%; Test for overall effect: Z = 2.04 (p = 0.04)

Forest plot demonstrating the meta-analysis of balance test with eyes closed between people with non-specific neck pain and healthy controls: The mean result is presented as confidence ellipse areas (CEA 95% bivariate ellipse); CI = Confident interval; HC = Healthy controls; n = Number of subjects; NSNP = Non-specific neck pain; SD = Standard deviation.

Study	HC Mean	SD	n	TNP Mean	SD	n	Hedges' g (CI95%)	Weight
Juul-Kristensen et al. 2013 (53)	653.5	285.96	10	1186	608.97	10	1.07 (0.15, 2.09)	20.33 %
Michaelson et al. 2003 (51)	1090	650	16	2690	1470	7	1.61 (0.64, 2.70)	17.47 %
De Pauw et al. 2018 (38)	176	61	30	411	288	35	1.08 (0.56, 1.62)	62.20 %
**Total**			**56**			**52**	**1.17 (0.14, 1.78)**	**100 %**

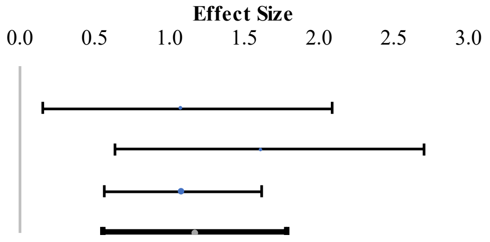

Heterogeneity: I2 = 0.00%; Test for overall effect: Z = 8.18 (p < 0.01)

Forest plot demonstrating the meta-analysis of balance test with eyes closed between people with traumatic neck pain and healthy controls: The mean result is presented as confidence ellipse areas (CEA 95% bivariate ellipse); CI = Confident interval; HC = Healthy controls; n = Number of subjects; SD = Standard deviation; TNP = Traumatic neck pain.

Study	HC Mean	SD	n	NSNP Mean	SD	n	Hedges' g (CI95%)	Weight
Jorgensen et al 2011 (52)	470	236	109	485	236	85	0.06 (-0.22, 0.35)	53.81 %
Lange et al 2014 (54)	425	223	25	485	276	30	0.23 (-0.30, 0.77)	22.29 %
Alizadeh et al 2022 (55)	353	346	30	262	155	30	-0.34 (-0.85, 0.17)	23.90 %
**Total**			**164**			**145**	**0.01 (-0.61, 0.62)**	**100 %**

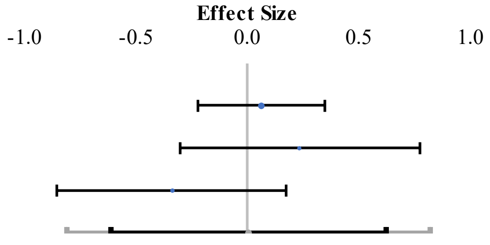

Heterogeneity: I2 = 24.15%; Test for overall effect: Z = 0.04 (p = 0.97)

Forest plot demonstrating the meta-analysis of balance test with eyes open between people with non-specific neck pain and healthy controls: The mean result is presented as confidence ellipse areas (CEA 95% bivariate ellipse); CI = Confident interval; HC = Healthy controls; n = Number of subjects; NSNP = Non-specific neck pain; SD = Standard deviation.

Study	HC Mean	SD	n	TNPD Mean	SD	n	Hedges' g (CI95%)	Weight
Dispenza et al. 2011 (50)	0.87	0.04	23	0.87	0.04	33	0.00 (-0.54, 0.54)	23.90 %
Prushansky et al. 2004 (48)	0.86	0.01	23	0.79	0.11	26	-0.86 (-1.46, -0.28)	22.24 %
Tjell et al. 1998 (11)	0.87	0.05	30	0.83	0.09	50	-0.51 (-0.98, -0.05)	26.21 %
Treleaven et al. 2005 (49)	0.88	0.07	50	0.81	0.07	50	-0.99 (-1.42, -0.58)	27.66 %
**Total**			**126**			**159**	**-0.60 (-1.30, 0.10)**	**100 %**

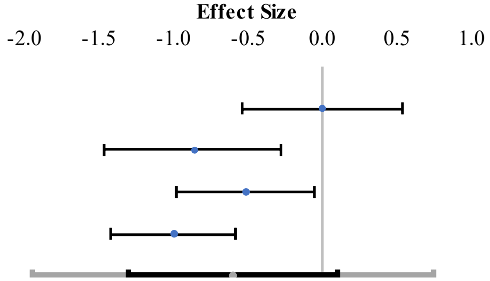

Heterogeneity: I2 = 67.78%; Test for overall effect: Z = -2.72 (p < 0.01)

Forest plot demonstrating the meta-analysis of oculomotor function between people with traumatic neck pain with dizziness and healthy controls: The mean result is presented as a mean gain; CI = Confident interval; HC = Healthy controls; n = Number of subjects; SD = Standard deviation; TNPD = Traumatic neck pain with dizziness.

Study	HC Mean	SD	n	TNPND Mean	SD	n	Hedges' g (CI95%)	Weight
Dispenza et al. 2011 (50)	0.87	0.04	23	0.87	0.04	33	0.00 (-0.54, 0.54)	24.33 %
Prushansky et al. 2004 (48)	0.86	0.01	23	0.79	0.11	26	-0.86 (-1.46, -0.28)	22.44 %
Tjell et al. 1998 (11)	0.87	0.05	30	0.85	0.06	25	-0.36 (-0.91, 0.17)	24.24 %
Treleaven et al. 2005 (49)	0.88	0.07	50	0.82	0.07	50	-0.85 (-1.27, -0.45)	28.99 %
**Total**			**126**			**134**	**-0.53 (-1.19, 0.13)**	**100 %**

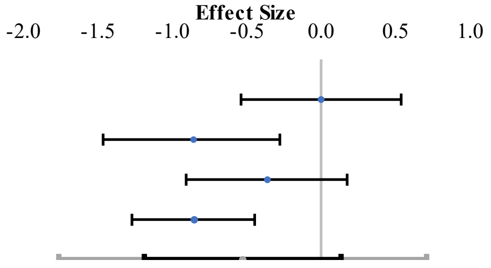

Heterogeneity: I2 = 61.97%; Test for overall effect: Z = -2.54 (p = 0.01)

Forest plot demonstrating the meta-analysis of oculomotor function between people with traumatic neck pain with non-dizziness and healthy controls: The mean result is presented as a mean gain; CI = Confident interval; HC = Healthy controls; n = Number of subjects; SD = Standard deviation; TNPND = Traumatic neck pain without dizziness.

	HC			TNPD				
**Study**	**Mean**	**SD**	**n**	**Mean**	**SD**	**n**	**Hedges' g (CI95%)**	**Weight**
Prushansky et al. 2004 (48)	0.035	0.097	23	0.026	0.125	26	-0.08 (-0.65, 0.49)	32.34 %
Tjell et al. 1998 (11)	0.02	0.02	30	0.14	0.14	50	1.06 (0.56, 1.56)	33.52 %
Treleaven et al. 2005 (49)	0.01	0.07	50	0.11	0.07	50	1.42 (0.99, 1.87)	34.14 %
**Total**			**103**			**126**	**0.82 (-1.12, 2.75)**	**100 %**

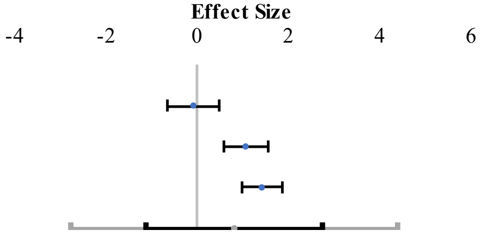

Heterogeneity: I2 = 88.84%; Test for overall effect: Z = 1.82 (p = 0.07)

Forest plot demonstrating the meta-analysis of oculomotor function between people with traumatic neck pain with dizziness and healthy controls: The mean result is presented as a difference between the gain in natural and the average values in the torsional position; CI = Confident interval; HC = Healthy controls; n = Number of subjects; SD = Standard deviation; TNPD = Traumatic neck pain with dizziness.

	HC			TNPND				
**Study**	**Mean**	**SD**	**n**	**Mean**	**SD**	**n**	**Hedges' g (CI95%)**	**Weight**
Prushansky et al. 2004 (48)	0.035	0.097	23	0.026	0.125	26	-0.08 (-0.65, 0.49)	32.05 %
Tjell et al. 1998 (11)	0.02	0.02	30	0.1	0.11	25	1.04 (0.49, 1.63)	31.88 %
Treleaven et al. 2005 (49)	0.01	0.07	50	0.07	0.07	50	0.85 (0.45, 1.27)	36.07 %
**Total**			**103**			**101**	**0.61 (-0.85, 2.08)**	**100 %**

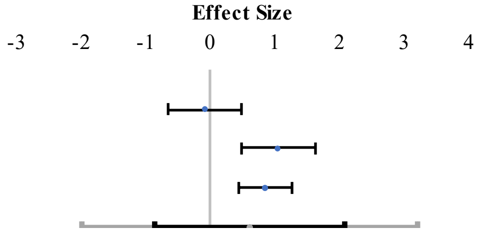

Heterogeneity: I2 = 79.03%; Test for overall effect: Z = 1.80 (p = 0.07)

Forest plot demonstrating the meta-analysis of oculomotor function between people with traumatic neck pain with non-dizziness and healthy controls: The mean result is presented as a difference between the gain in natural and the average values in the torsional position; CI = Confident interval; HC = Healthy controls; n = Number of subjects; SD = Standard deviation; TNPND = Traumatic neck pain without dizziness.

Appendix 4: Detailed evaluation of the risk of bias of the included studies according to QUADAS-2

Study	Risk of bias				Applicability concerns		
	Patient selection	Index test(s)	Reference standard	Flow and timing	Patient selection	Index test(s)	Reference standard
**Joint position sense**							
**Alalawi et al. 2022 (44)**	High[e,m]	Unclear[j]	Unclear[b]	Unclear[d,i]	Low	Unclear	Low
**Alalawi et al 2022 (45)**	High[h,m]	Unclear[j]	Unclear[b]	Unclear[d]	Low	Low	Low
**Cheever et al. 2017 (37)**	High[a,m]	Low	Unclear[b]	Unclear[d]	Low	Low	Low
**Cid et al. 2022 (46)**	High[h,m]	High[c]	Unclear[b]	High[l]	Low	Low	Low
**Dugailly et al. 2015 (36)**	High[m]	Unclear[j]	Unclear[b]	Unclear[d,i]	Low	Low	Low
**Elsig et al. 2014 (35)**	High[e,m]	Unclear[j]	Low	Low	Unclear	Unclear	Low
**Ghamkhar et al. 2020 (41)**	High[m]	Unclear[j]	Low	Low	Low	Unclear	Low
**Goncalves et al. 2019 (40)**	High[e,m]	High[c]	Low	Unclear[d,i]	Unclear	High	Low
**Grip et al. 2007 (29)**	High[m]	Unclear[f]	Unclear[b]	High[g]	Low	Unclear	Unclear
**Heikkilä et al. 1998 (25)**	High[m]	Unclear[j]	Unclear[b]	Low	Low	Low	Low
**Hill et al. 2009 (31)**	High[a,m]	Unclear[k]	Unclear[b]	High[g]	Low	Low	Low
**Kristjansson et al. 2003 (26)**	High[m]	Low	Unclear[b]	Low	Low	Low	Unclear
**Lopez-de-Uralde-Villanueva et al. 2020 (42)**	High[m]	Low	Low	Low	Low	Low	Low
**Micarelli et al. 2020 (43)**	High[m]	Unclear[j]	Unclear[b]	Low	Low	Unclear	Low
**Moustafa et al. 2022 (47)**	High[m]	Unclear[j]	Unclear[b]	Low	Low	Low	Low
**Van den Oord et al. 2010 (33)**	High[m]	Low	Unclear[b]	Unclear[d]	High	Low	Low
**De Pauw et al. 2018 (38)**	High[a,m]	Low	Unclear[b]	Low	High	Low	Low
**Portelli et al. 2018 (39)**	High[m]	Unclear[j]	Unclear[b]	Unclear[d,i]	Low	Unclear	Low
**Revel et al. 1991 (24)**	High[e,m]	Unclear[j]	Unclear[b]	Low	Unclear	Unclear	Unclear
**Roren et al. 2009 (32)**	High[h,m]	Low	Low	Unclear[d,i]	Low	Low	Low
**Sterling et al. 2003 (27)**	High[m]	Unclear[j]	Unclear[b]	Unclear[d]	Low	Low	Low
**Sterling et al. 2004 (28)**	High[m]	Low	Unclear[b]	Unclear[d]	Low	Low	Low
**Treleaven et al. 2003 (12)**	High[e,m]	Low	Low	Low	Unclear	Low	Low
**Uthaikhup et al. 2012 (34)**	High[h,m]	Unclear[j]	Unclear[b]	Low	Low	Low	Low
**Woodhouse et al. 2008 (30)**	High[m]	High[c]	Unclear[b]	Low	Low	High	Low
**Oculomotor function**							
**Dispenza et al. 2011 (50)**	High[m]	Unclear[j]	Unclear[b]	Unclear[i]	Low	Low	Low
**Prushansky et al. 2004 (48)**	High[m]	Unclear[j]	Unclear[b]	Unclear[d,i]	Low	Low	Low
**Tjell et al. 1998 (11)**	High[m]	Low	Unclear[b]	Low	Low	Low	Unclear
**Treleaven et al. 2005 (49)**	High[m]	Low	Unclear[b]	Low	Low	Low	Unclear
**Balance**							
**Alizadeh et al 2022 (55)**	High[m]	Low	Unclear[b]	Low	Low	Low	Low
**Jorgensen et al. 2011 (52)**	High[e,m]	Low	Unclear[b]	Unclear[d,i]	Unclear	Low	Low
**Juul-Kristensen et al. 2013 (53)**	High[m]	Unclear[j]	Unclear[b]	Unclear[d]	Low	Low	Unclear
**Lange et al. 2014 (54)**	High[e,m]	Low	Unclear[b]	Low	Low	Low	Low
**Michaelson et al. 2003 (51)**	High[m]	Low	Unclear[b]	Low	Low	Low	Low
**De Pauw et al. 2018 (38)**	High[a,m]	Low	Unclear[b]	Low	High	Low	Low

a = Inappropriate inclusion or exclusions identified; b = Reference standard remains unclear; c = Testers were not blinded to group allocation; d = Unclear if all participants assessed with the reference test; e = Ambiguity in the inclusion or exclusion criteria or enlistment; f = Ambiguity about the reliability of instrument used; g = Inappropriate test flow identified; h = Identified inappropriate enlistment (e.g. preselection of subjects); i = The testing flow remained unclear (time interval unclear); j = Ambiguity about the testers´ blinding to group allocation; k = Ambiguity of test implementation; l = The number of subjects fell short of the calculated sample size; m = Case-control design was not avoided
